# Random Projection for Fast and Efficient Multivariate Correlation Analysis of High-Dimensional Data: A New Approach

**DOI:** 10.3389/fgene.2016.00102

**Published:** 2016-06-07

**Authors:** Claudia Grellmann, Jane Neumann, Sebastian Bitzer, Peter Kovacs, Anke Tönjes, Lars T. Westlye, Ole A. Andreassen, Michael Stumvoll, Arno Villringer, Annette Horstmann

**Affiliations:** ^1^Department of Neurology, Max Planck Institute for Human Cognitive and Brain SciencesLeipzig, Germany; ^2^IFB Adiposity Diseases, Leipzig University Medical CenterLeipzig, Germany; ^3^Collaborative Research Center 1052-A5, University of LeipzigLeipzig, Germany; ^4^Department of Psychology, Dresden University of TechnologyDresden, Germany; ^5^Hospital for Endocrinology and Nephrology, University Hospital LeipzigLeipzig, Germany; ^6^Norwegian Centre for Mental Disorders Research (NORMENT), KG Jebsen Centre for Psychosis Research, University Hospital OsloOslo, Norway; ^7^Department of Psychology, University of OsloOslo, Norway; ^8^Clinic for Cognitive Neurology, University Hospital LeipzigLeipzig, Germany; ^9^Mind and Brain Institute, Berlin School of Mind and Brain, Humboldt-University and CharitéBerlin, Germany

**Keywords:** multivariate multimodal data integration, Partial Least Squares Correlation, dimensionality reduction, genome-wide association, genetic neuroimaging

## Abstract

In recent years, the advent of great technological advances has produced a wealth of very high-dimensional data, and combining high-dimensional information from multiple sources is becoming increasingly important in an extending range of scientific disciplines. Partial Least Squares Correlation (PLSC) is a frequently used method for multivariate multimodal data integration. It is, however, computationally expensive in applications involving large numbers of variables, as required, for example, in genetic neuroimaging. To handle high-dimensional problems, dimension reduction might be implemented as pre-processing step. We propose a new approach that incorporates Random Projection (RP) for dimensionality reduction into PLSC to efficiently solve high-dimensional multimodal problems like genotype-phenotype associations. We name our new method PLSC-RP. Using simulated and experimental data sets containing whole genome SNP measures as genotypes and whole brain neuroimaging measures as phenotypes, we demonstrate that PLSC-RP is drastically faster than traditional PLSC while providing statistically equivalent results. We also provide evidence that dimensionality reduction using RP is data type independent. Therefore, PLSC-RP opens up a wide range of possible applications. It can be used for any integrative analysis that combines information from multiple sources.

## 1. Introduction

The majority of human neurological and psychiatric disorders are substantially heritable (Plomin et al., 1994; Meyer-Lindenberg and Weinberger, 2006; Bigos and Weinberger, 2010; Ge et al., 2013). Since these illnesses represent an actual problem of public health, it is vitally important to understand the underlying genetic mechanisms. Substantial progress has been achieved in recent years with the emergence of genome-wide association (GWA) studies (Haines et al., 2005). Typically, these studies focus on single-nucleotide polymorphisms (SNPs), the most common type of human genetic variation (Wang et al., 1998; Crawford and Nickerson, 2005). However, the identification of new SNPs in GWA studies does not necessarily reveal the variations that also contribute to human diseases. The discovery of the biological function that arises from these DNA sequence variations requires the investigation of the complex relationship between genotype and phenotype information (Pevsner, 2009; 1000 Genomes Project Consortium et al., 2010; Baker, 2012), which is known as the intermediate phenotype concept (Gottesman and Shields, 1967; Gottesman and Gould, 2003). For a number of human neurological and psychiatric disorders, in particular, alterations in brain anatomy, function and connectivity have been shown to be highly heritable and reliably correlated with the disease (Jansen et al., 2015). Consequently, measures derived from *in-vivo* anatomical or functional neuroimaging were increasingly introduced as intermediate phenotypes for genetic association analyses.

The statistical analysis of the relationship between the SNP and the neuroimaging measures requires the solution of high-dimensional association problems. Since both data sources naturally involve large numbers of variables, computationally efficient analysis frameworks are pivotal. Multivariate statistical techniques have commonly been used in this field, since they are able to combine the information from multiple markers and multiple sources simultaneously into the analysis. However, the higher the dimensionality, the more challenging is the analysis from a statistical and computational point of view. This relates to a phenomenon known as the curse of dimensionality, which states that obtaining a statistically reliable result requires the sample size to grow exponentially with the dimension (Bellman, 1957, 1960). In addition, computation times become excessively long with increasing data dimensionality, posing a serious practical problem for many applications.

One approach for mitigating high-dimensional data analysis is dimensionality reduction. Various dimensionality reduction techniques have been proposed for pre-processing in genetic neuroimaging. In a whole-genome setup including 85,772 SNPs and 34 brain locations of interest, Le Floch et al. (2012) used univariate filters with different thresholds in order to identify a subset of SNPs that were considerably correlated to the neuroimaging data. The reduced set of SNPs was further searched for association with the neuroimaging data using two multivariate strategies, penalized Partial Least Squares regression (Wold, 1975) and regularized Kernel Canonical Correlation Analysis (Hotelling, 1936). The authors showed that a relatively large number of SNPs was needed after filtering in order to comprise all true positives. However, to avoid over-fitting, irrelevant SNPs had to be filtered out although the authors did not define a clear threshold for the filters. In addition to univariate filtering, Le Floch et al. (2012) applied Principal component analysis (PCA) (Pearson, 1901) for dimensionality reduction. Specifically, they performed PCA on both the neuroimaging and the SNP data set and kept as many components as necessary to explain 99% of the variance in each modality. However, all methods based on PCA failed to identify generalizable associations.

PCA-based dimensionality reduction was also conducted in a study by Hibar et al. (2011a,b). The authors searched for associations between 448,293 genome-wide SNPs and 31,662 whole-brain voxels in a large sample of 731 subjects from the Alzheimer's disease neuroimaging initiative (ADNI). To reduce the total number of tests, SNPs were grouped into 18,044 genes based on gene membership. Principal component regression was then implemented to search for the combined effect of multiple SNPs on the brain. Hibar et al. (2011a,b) named their technique voxel-wise gene-wide association study (vGeneWAS). However, no genes identified were significant after correction for multiple testing.

Using the same data set of genome-wide SNPs and whole-brain neuroimaging voxels from the ADNI, in a recent study, Hua et al. (2015) performed dimensionality reduction by selecting 119 brain regions of interest based on an anatomical brain atlas. Distance covariance (Székely et al., 2007) was applied to infer the relationship between the single SNP predictors from the entire genome and the average voxel values at the 119 brain regions, utilized as multivariate response. In order to overcome the multiple testing problem, Hua et al. (2015) also introduced a local false discovery rate (FDR) modeling algorithm. The authors showed that using their method, they were able to find 23,128 significant SNPs at α-level 0.05, while simple linear regression yielded no significant SNPs.

As previous studies have shown, dimensionality reduction using univariate filters or PCA solves critical over-fitting issues in genetic neuroimaging applications (Le Floch et al., 2012). However, both strategies present major limitations. As discussed by Le Floch et al. (2012), univariate filters are not the optimal choice, since they can not account for interdependencies between variables. The genetic and the neuroimaging variants are, however, naturally highly collinear. PCA has two major limitations. First, PCA explicitly depends on the input data, since it uses an optimization criterion to transform the original variables into a set of orthogonal principal components (Goel et al., 2005; Sulić et al., 2010). Second and most importantly, it is computationally expensive, since its runtime is quadratic in the number of dimensions (Menon, 2007). Thus, in genetic neuroimaging, where the data sets may capture the whole brain and the whole genome, respectively, PCA is infeasible in case there is no powerful compute server available in the lab.

A dimensionality reduction technique that is computationally efficient is Random projection (RP) (Johnson and Lindenstrauss, 1984). RP uses a random matrix with unit Euclidean column norms to find a lower-dimensional subspace that approximately preserves the distances between all pairs of data points in the original space (Kaski, 1998; Dasgupta, 2000; Bingham and Mannila, 2001; Lin and Gunopulos, 2003; Vempala, 2004). In a number of data mining and biological studies (Papadimitriou et al., 1998; Bingham and Mannila, 2001; Goel et al., 2005; Sulić et al., 2010; Liu and Fieguth, 2012; Palmer et al., 2015), RP has been shown to provide good results. Several studies also compared RP and PCA and showed that their overall performance was comparably similar, while RP had much lower computational requirements (e.g., Bingham and Mannila, 2001; Goel et al., 2005).

In this study, we propose a new approach that uses RP for dimensionality reduction to efficiently assess the relationship between genetic variation and brain imaging measures as an example for high-dimensional genotype-phenotype association problems. In particular, we consider Partial Least Squares Correlation (PLSC) (McIntosh et al., 1996; Tucker, 1958), which has been shown to be most appropriate for combined analysis of SNP and neuroimaging data in a systematic comparison of multivariate techniques (Grellmann et al., 2015). A key limitation of current PLSC implementations is that they are computationally expensive in handling large numbers of variables. We therefore incorporate RP into PLSC, and name our new method PLSC-RP. Using both simulated and experimental data sets containing whole genome SNPs and whole brain neuroimaging measures, we demonstrate that PLSC-RP provides statistically equivalent results to traditional PLSC. However, using PLSC-RP, runtime is reduced from hours to seconds. We also provide evidence that dimensionality reduction using RP is data type independent and can thus be applied to both continuous and count data. To establish RP for multivariate analysis of high-dimensional genotype-phenotype-associations is, to our knowledge, a strategy never used before. Importantly, compared to previous applications of RP in data mining and biological studies (Papadimitriou et al., 1998; Bingham and Mannila, 2001; Goel et al., 2005; Sulić et al., 2010; Liu and Fieguth, 2012; Palmer et al., 2015), we had to address two additional problems. First, in order to be able to evaluate the contribution of a single genotypic or phenotypic variable, the multivariate associations detected by PLSC-RP must be interpretable in the original spaces after dimensionality reduction. In contrast, previous applications exploited RP such that learning, classification or clustering were carried out directly in the compressed domain (e.g., Liu and Fieguth, 2012; Palmer et al., 2015). Second, in genotype-phenotype association studies, both the genetic and the phenotypic data, are naturally high-dimensional. Thus, RP needs to be applied for dimensionality reduction in both domains. The PLSC-RP method we introduce in this study is favorable since it fulfills both requirements: the back-transformation of results after dimensionality reduction is straightforward, and it can be applied to reduce the number of both the genetic and phenotypic variables.

In this study, we explicitly applied PLSC-RP for efficient assessment of genome-wide and whole-brain relations as an example for high-dimensional association problems. However, the application of PLSC-RP is not limited to the combined analysis of brain imaging and genotype data. It might be considered for fusion of multimodal biological assays such as genomic, transcriptomic and proteomic data, for fusion of multimodal brain imaging techniques or, in epidemiological research, for fusion of environmental factors and measures characterizing health status. Since PLSC-RP depends on sample size only and is independent of the number of variables, it is especially attractive for large-scale multicenter association studies or other data sharing projects.

## 2. Materials and methods

### 2.1. Random projection

Random Projection (RP) is a dimensionality reduction technique, which uses a random matrix with unit Euclidean column norms to find a lower-dimensional subspace that approximately preserves the distances between all pairs of data points in the original space (Kaski, 1998; Dasgupta, 2000; Bingham and Mannila, 2001; Lin and Gunopulos, 2003; Vempala, 2004). The concept of RP is as follows: Given a data matrix ***X*** ∈ ℝ^*N*×*d*^, where *N* is the total number of points and *d* is the original dimension, RP transforms ***X*** to a lower dimensional space via the transformation:

(1) $$\begin{array}{l} X_{\text{RP}}=X\cdot R, \end{array}$$

where ***R***∈ℝ^*d*×*k*^ is a random matrix with unit Euclidean column norms and $X_{\text{RP}}\in {\mathbb{R}}^{N\times k}$ is the low-dimensional subspace with desired lower dimension *k*. The basic idea for RP is derived from the Johnson-Lindenstrauss lemma (Johnson and Lindenstrauss, 1984), which states that a set of *N* points in a high-dimensional space can be mapped onto a $k>k_{0}=O(\frac{\log\left(N\right)}{\epsilon^{2}})$ dimensional subspace such that the distances between the points are approximately preserved, i.e., not distorted more than by a factor of 1±ϵ, for any ϵ>0. Note that the dimensionality reduction according to Johnson and Lindenstrauss (1984) depends on the number of points *N* only, since *k* is logarithmic in *N* and independent of the original dimension *d*. A proof of this lemma was provided by several authors (Frankl and Maehara, 1988; Indyk and Motwani, 1998; Dasgupta and Gupta, 1999).

### 2.2. Partial least squares correlation

PLSC (McIntosh et al., 1996), first introduced as Tucker Inter-battery Analysis (Tucker, 1958), is a correlation technique that analyzes the association between two sets of variables $X_{1}\in {\mathbb{R}}^{N\times d_{1}}$ and $X_{2}\in {\mathbb{R}}^{N\times d_{2}}$. In our application, Matrix ***X*_*1*_** collects in each column the phenotype measures, e.g., the brain activity at each voxel in the brain. Matrix ***X*_*2*_** stores the genotype measures in each column, e.g., the number of minor alleles for a given SNP. The number of rows corresponds to the sample size.

To model the relationship between ***X*_*1*_** and ***X*_*2*_**, which are both standardized column-wise, PLSC successively builds orthogonal linear combinations of the observed variables of each set (so-called latent variables), such that the covariance between the pair of latent variables is maximized,

(2) $$\begin{array}{l} \underset{|w_{1_{i}}|=|w_{2_{i}}|=1}{\max}\text{cov}\left(X_{1}w_{1_{i}},X_{2}w_{2_{i}}\right), \end{array}$$

where *i* = 1, …, *p*, *p* = min(*d*_1_, *d*_2_). The relationship between the columns of ***X*_*1*_** and ***X*_*2*_** is stored in a cross-product matrix $A=X_{1}^{{}^{\prime }}X_{2}$. Singular Value Decomposition (SVD) is used to decompose *A* into three matrices,

(3) $$\begin{array}{l} A=X_{1}^{\prime }X_{2}=USV^{\prime }=s_{1}u_{1}v_{1}^{\prime }+s_{2}u_{2}v_{2}^{\prime }+\ldots +s_{p}u_{p}v_{p}^{\prime }, \end{array}$$

where *p* = min(*d*_1_, *d*_2_). The coefficients of the PLSC, ***W*_*1*_** = [***w*_*1*_*1*__**, …, ***w*_*1*_*p*__**] and ***W*_*2*_** = [***w*_*2*_*1*__**, …, ***w*_*2*_*p*__**], equal the matrices of left and right singular vectors ***U*** and ***V***. They describe the contribution of each variable in ***X*_*1*_** and ***X*_*2*_** to the construction of the linear combinations ***Z*_*1*_** = ***X*_*1*_*W*_*1*_** and ***Z*_*2*_** = ***X*_*2*_*W*_*2*_**, so-called latent variables or scores. The singular values *s*_*i*_, *i* = 1, …, *p*, in the SVD provide the covariance between the latent variables.

### 2.3. Random projection for dimensionality reduction in partial least squares correlation

Assume that $X_{1}\in {\mathbb{R}}^{N\times d_{1}}$ and $X_{2}\in {\mathbb{R}}^{N\times d_{2}}$ are two data sets, where *N* is the sample size and *d*_1_ and *d*_2_ are the number of variables in ***X*_*1*_** and ***X*_*2*_**, respectively. Both ***X*_*1*_** and ***X*_*2*_** are column-wise standardized. When the number of columns in ***X*_*1*_** and ***X*_*2*_** is high, PLSC will be computationally expensive. To address the association between ***X*_*1*_** and ***X*_*2*_** using a computationally more efficient approach, we apply RP for dimensionality reduction prior to PLSC. We denote the PLSC analysis after dimensionality reduction using RP as PLSC-RP analysis. RP can be used to reduce the number of variables in ***X*_*1*_** (i.e., the phenotype measures), to reduce the number of variables in ***X*_*2*_** (i.e., the genotype measures), or both. Therefore, we multiply the high-dimensional matrices ***X*_*1*_** and/or ***X*_*2*_** with orthonormal random matrices ***R*_*1*_** and/or ***R*_*2*_**. We generate the elements of the random matrices according to the following algorithm (Dasgupta, 2000):

1. Assign to each entry of the matrix an independently and identically distributed (i.i.d.) N(0, 1) value.
2. Orthogonalize the *k* columns of the matrix using the Gram-Schmidt algorithm (Björck, 1967).
3. Normalize the columns of the matrix to unit length.

Orthogonalization is important to preserve distances between the original points in the low-dimensional space (Kaski, 1998). Unfortunately, enforcing the random matrices to be orthogonal requires the Gram–Schmidt algorithm, which is computationally expensive (Sulić et al., 2010). However, it has been shown that in high-dimensional spaces, there exists a much larger number of nearly orthogonal than truly orthogonal vectors (Hecht-Nielsen, 1994). Thus, high-dimensional random matrices might be sufficiently close to orthogonal (Lin and Gunopulos, 2003) and the orthogonalization step might be avoided without affecting the distance preserving properties of Random Projections. Note that two simpler algorithms for generating sparse random matrices have been proposed (Achlioptas, 2001). Multiplying the input matrices with sparse rather than dense random matrices increases multiplication efficiency. For proof of concept of PLSC-RP, however, we decided to generate the elements of the random matrices by a standard normal distribution (Dasgupta, 2000). This way, we avoided potential inaccuracies in our results due to sparsity, which we later cannot evaluate when comparing PLSC-RP to its state-of-the-art counterpart PLSC. We modified the algorithm by Dasgupta (2000) by omitting the orthogonalization using the Gram-Schmidt algorithm, since in our application random matrices were consistently high-dimensional.

### 2.4. PLSC-RP for dimensionality reduction in *X*_1_ or *X*_2_

Assume that ***X*_*1*_** is high-dimensional. RP transforms ***X*_*1*_** to a lower dimensional space via the following transformation:

(4) $$\begin{array}{l} X_{1_{\text{RP}}}=X_{1}\cdot R, \end{array}$$

where $R\in {\mathbb{R}}^{d_{1}\times k}$ is a random matrix and $X_{1_{\text{RP}}}\in {\mathbb{R}}^{N\times k}$ is the low-dimensional subspace of ***X*_*1*_** with desired lower dimension *k*. We determine the dimensionality of the low-dimensional subspace matrix *X*_*1*_RP__ according to the Menon theorem (Menon, 2007), which guarantees the preservation of pairwise distances with probability of at least 1−*N*^−β^ if $\epsilon \in \left[\frac{3}{4},\frac{3}{2}\right]$ for

(5) $$\begin{array}{l} k\geq k_{0}:=\frac{16+8\beta }{\epsilon^{2}}\cdot \log\left(N\right). \end{array}$$

For our application, we selected ϵ = 1.0 and 1−*N*^−β^ = 0.95 (yielding β = 0.6505). Note that for the accuracy of the distance preservation ϵ also values $\epsilon <\frac{3}{4}$ or $\epsilon >\frac{3}{2}$ are possible. However, for $\epsilon \in \left[\frac{3}{4},\frac{3}{2}\right]$, the Menon bound adapts best to the lowest reduced dimension of the projection. Accordingly, the term PLSC-RP denotes the process of building latent variables ***z***_***1***_*RP*_***i***___ = ***X***_***1***_RP__·***w***_***1***_*RP*_***i***___ and ***z***_***2***_***i***__ = ***X*_*2*_**·***w*_*2*_*i*__**, such that the covariance between the pair of latent variables ***z***_***1***_*RP*_***i***___ and ***z***_***2***_ is maximized,

(6) $$\begin{array}{l} \underset{|w_{1_{{\text{RP}}_{i}}}|=|w_{2_{i}}|=1}{\max}\text{cov}\left(z_{1_{{\text{RP}}_{i}}},z_{2_{i}}\right), \end{array}$$

where *i* = 1, …, *p*, *p* = min(*k, d*_2_).

According to Equation (6), we obtain the weights ***W*_*2*_** for data set ***X*_*2*_**, which are approximations for the weights computed using traditional PLSC, since ***X***_***1***_RP__ is only a compressed representation of ***X*_*1*_**. However, for the reduced data set, we get weights ***W***_***1***_RP__ of the low-dimensional subspace *X*_*1*_RP__. To evaluate the contribution of each single variable in ***X*_*1*_**, we transform the weights ***W***_***1***_RP__ back to the original space, that is ***W*_*1*_**. Since the weights ***W*_*2*_** obtained by SVD on the cross-product matrix $X_{1_{\text{RP}}}^{\prime }X_{2}$ are approximations for the weights obtained by performing SVD on $X_{1}^{\prime }X_{2}$, we obtain the original weights ***W*_*1*_** by inserting the weights ***W*_*2*_** into the equation for the SVD and rearranging it as follows:

(7) $$\begin{array}{l} w_{1_{i}}=\frac{1}{s_{i}\cdot |w_{2_{i}}{|}^{2}}\cdot A\cdot w_{2_{i}}, \end{array}$$

where $A=X_{1}^{\prime }X_{2}$ is the cross-product matrix, *s*_*i*_ is the singular value of component *i*, *i* = 1, …, *p*, *p* = min(*k, d*_2_), when performing SVD based on $X_{1_{\text{RP}}}^{\prime }X_{2}$, and component *i* is the component explaining the largest proportion of summed squared cross-block correlations (Bookstein, 1994; McIntosh et al., 1996). The derivation of Equation (7) is embodied in the Supplementary Equations (1)–(3). Applying RP to reduce the number of variables in ***X*_*2*_** follows the same logic.

PLSC-RP will be significantly faster than traditional PLSC, since it operates on a matrix with a much smaller number of columns ($X_{1_{\text{RP}}}\in {\mathbb{R}}^{N\times k}$ rather than $X_{1}\in {\mathbb{R}}^{N\times d_{1}}$ with *k*≪*d*_1_). Importantly, permutation testing (Edgington, 1980) can also be performed on the low-dimensional space.

### 2.5. PLSC-RP for dimensionality reduction in ***X*_*1*_** and ***X*_*2*_**

Assume that both ***X*_*1*_** and ***X*_*2*_** are high-dimensional. RP transforms ***X*_*1*_** and ***X*_*2*_** to lower dimensional spaces via the following transformation:

(8) $$\begin{array}{r} \begin{array}{ccc} X_{1_{\text{RP}}} & = & X_{1}\cdot R_{1},\\ X_{2_{\text{RP}}} & = & X_{2}\cdot R_{2}. \end{array} \end{array}$$

We determine the dimensionality of ***X***_***1***_RP__ and ***X***_***2***_RP__ according to Menon (2007). Note that if ***X*_*1*_** and ***X*_*2*_** have the same number of variables, it is sufficient to generate a joint random matrix ***R*** for transformation. In general, however, the number of variables differs between ***X*_*1*_** and ***X*_*2*_**.

Subsequently, we apply PLSC in order to successively build latent variables ***z***_***1***_*RP*_*i*___ = ***X***_***1***_RP__ · ***w***_***1***_*RP*_***i***___ and ***z***_***2***_*RP*_*i*___ = ***X***_***2***_RP__ · ***w***_***2***_*RP*_*i*___, such that the covariance between the pair of latent variables ***z***_***1***_*RP*_*i*___ and ***z***_***2***_*RP*_*i*___ is maximized, *i* = 1, …, *p*, *p* = min(*k*_1_, *k*_2_). Since the dimensionality in *****X***_***1***_** and ***X*_*2*_** is reduced by RP, we obtain weights ***W***_***1***_RP__ and ***W***_***2***_RP__ for the low dimensional subspaces ***X***_***1***_RP__ and ***X***_***2***_RP__. To evaluate the contribution of each single variable in *****X***_***1***_** and ***X*_*2*_**, we transformed the weights ***W***_***1***_RP__ and ***W***_***2***_RP__ back to the original space, that is ***W*_*1*_** and ***W*_*2*_**, as follows:

(9) $$\begin{array}{r} \begin{array}{ccc} w_{1_{i}} & = & \frac{1}{s_{i}\cdot |w_{2_{{\text{RP}}_{i}}}{|}^{2}}\cdot \text{cov}\left(X_{1},X_{2_{\text{RP}}}\right)\cdot w_{2_{{\text{RP}}_{i}}},\\ w_{2_{i}} & = & \frac{1}{s_{i}\cdot |w_{1_{{\text{RP}}_{i}}}{|}^{2}}\cdot {\left(\text{cov}\left(X_{1_{\text{RP}}},X_{2}\right)\right)}^{\prime }\cdot w_{1_{{\text{RP}}_{i}}}, \end{array} \end{array}$$

where *s*_*i*_ is the singular value of component *i*, *i* = 1, …, *p*, *p* = min(*k*_1_, *k*_2_), when performing SVD based on $X_{1_{\text{RP}}}^{\prime }X_{2_{\text{RP}}}$, and component *i* is the component explaining the largest proportion of summed squared cross-block correlations (Bookstein, 1994; McIntosh et al., 1996). The derivation of Equation (10) is again elucidated in the Supplementary Material (Supplementary Equations 4–6).

### 2.6. Comparison of PLSC and PLSC-RP weight profiles

We evaluate the performance of PLSC-RP by comparing its weight profiles to the weight profiles of PLSC applied to the same original data set. To measure similarity, we consider three similarity measures: Pearson correlation (Anderberg, 1973), the cosine measure (Anderberg, 1973) and the extended Jaccard similarity (Strehl and Ghosh, 2000). In addition, we used ANOVA (Chambers et al., 1992) for comparison. The ANOVA model represents a perfect linear relationship between the weights of PLCS and PLSC-RP, in case the intercept equals 0 and the slope equals 1.

### 2.7. Permutation testing

Permutation testing is used to assess the significance of the covariance between the pair of latent variables

- ***z***_***1***_***i***__ and ***z***_***2***_***i***__, *i* = 1, …, *p*, *p* = min(*d*_1_, *d*_2_) (traditional PLSC),
- ***z***_***1***_*RP*_***i***___ and ***z***_***2***_***i***__, *i* = 1, …, *p*, *p* = min(*k, d*_2_) (PLSC-RP on ***X***_***1***_RP__ and ***X*_*2*_**),
- ***z***_***1***_*RP*_***i***___ and ***z***_***2***_*RP*_***i***___, *i* = 1, …, *p*, *p* = min(*k*_1_, *k*_2_) (PLSC-RP on ***X***_***1***_RP__ and ***X***_***2***_RP__),

respectively. For this purpose, observations, i.e., rows of input matrices, are randomly reassigned without replacement and PLSC (PLSC-RP, respectively) are recalculated. At each permutation, the statistic (i.e., the covariance of latent variables) is then compared to the statistic obtained on the original data with probability value equal to the number of times the statistic of permuted data exceeds the original value.

### 2.8. Data sets

#### 2.8.1. PLSC-RP for high-dimensional phenotype measures derived from neuroimaging

First we illustrate the PLSC-RP methodology for association analysis of two data sets, where one contains high-dimensional brain imaging variables as phenotypes. As an example, we used functional magnetic resonance imaging (fMRI) data, which has been shown to be highly heritable and reliably correlated with a number of human neurological and psychiatric diseases (Jansen et al., 2015). Functional MRI is a brain imaging technique for measuring neural activity based on changes in blood oxygenation and blood flow. The brain activity is accessed in volume pixel elements, called voxels, of the three-dimensional functional magnetic resonance image. Specifically, the activity in a voxel is defined as how closely the time-course of the signal from that voxel matches the expected time-course of activation, which is determined by the experimental design (Barad et al., 2009).

As simulation example, we generated fMRI data of increasing dimensionality (1000, 10,000, 20,000, 30,000, 40,000, 50,000, 70,000, and 90,000 voxels) using multivariate normal distribution with mean and covariance parameters estimated from experimental fMRI contrast images (Le Floch et al., 2012). We searched for associations with 50 candidate SNPs simulated using the gs algorithm (Li and Chen, 2008) based on phase III HapMap data (The HapMap Consortium, 2003). SNPs were recoded using the additive genetic model, counting the number of minor alleles per person. The sample size was chosen to be 100 for all data matrices. For each simulated dimensionality of the fMRI data set, we induced a linear relationship between one randomly selected voxel and three SNPs, such that the pairwise correlation between that voxel, voxels in collinearity with the selected voxel and the selected SNPs was on average 0.3. This is in line with association strengths reported in other studies (Filippini et al., 2009; Potkin et al., 2009; Ousdal et al., 2012).

Using our simulated data, we first applied PLSC to calculate voxel and SNP weights that maximize the covariance between the two data sets. We computed as many components as necessary to explain at least 80% of variance and defined the causal component, that is the component comprising the linear relationship between selected voxels and SNPs, by the out-of-sample covariance, estimated using 10-fold cross-validation (Le Floch et al., 2012). To assess the significance of the covariance and to quantify the reliability of weights, we used permutation testing (Edgington, 1980) and bootstrapping (Tibshirani and Efron, 1993), respectively.

Next, we applied PLSC-RP in order to optimize runtime. As we searched for linear relations between high-dimensional fMRI measures and a comparably small number of SNPs, we applied RP to reduce the number of voxels and kept the raw SNP matrix. To determine the dimensionality of the low-dimensional fMRI subspace matrix, we adopted the Menon theorem (Menon, 2007) and reduced the number of voxels to 100 dimensions (Menon lower bound equaled *k* = 97.6486) for all considered dimensionalities of the fMRI data set. After back-transformation, we compared the weight profiles of PLSC-RP to the weight profiles obtained using traditional PLSC on the same original data set.

In order to verify our findings on simulated data, we considered an experimental brain imaging genetics data set that has been published previously (Ousdal et al., 2012) to test the hypothesis that monoamines are important modulators of amygdala activity in the brain (LeDoux, 2007). Therefore, the authors combined whole-genome microarray SNPs with fMRI data collected during emotional face-matching task, in which the participants (healthy controls and patients with diagnosis of schizophrenia, bipolar disorder or other psychosis) were presented with two stimuli (human faces expressing anger of fear) that had to be matched to a target stimulus (Hariri et al., 2002; Carre et al., 2010). Genotyping was done using an array-based whole-genome assay and SNPs were recoded using an additive genetic model. The original analysis was performed using SPM2 (Friston et al., 2007) following standard pre-processing pipelines for fMRI data and controlling for diagnosis type. Gender and age were not significantly different across subject groups. To search for genome-wide SNPs modulating amygdala activity, individual contrast values for the right and left amygdala peak voxel were tested for association with each SNP separately. The authors reported a significant association between activation of the left amygdala peak voxel and three SNPs in high linkage disequilibrium, namely rs10014254 (*P* = 4.16 × 10^−8^), rs11722038 (*P* = 4.20 × 10^−8^) and rs17529323 (*P* = 4.66 × 10^−8^). A significant interaction between SNP and diagnosis type was not reported (*P* = 0.28). A more detailed description of recruitment, experimental task, fMRI data acquisition, genotyping, quality control and statistical analysis is provided in the original publication (Ousdal et al., 2012).

To demonstrate the PLSC-RP methodology, we used updated fMRI images that have been preprocessed using FSL software (Smith et al., 2004) and genotype information on five SNPs (rs10014254, rs11722038, rs17529323, rs382013, and rs437633). Instead of focusing on amygdala as region of interest as in the original study, we considered whole-brain measures in order to detect further brain regions involved in the face-matching task. After removing missing data for all SNPs, we were left with 208 subjects. Statistical analysis was performed as follows. At first we corrected for diagnosis type as in the original publication. Then, we applied PLSC considering whole-brain voxels. Finally, we used our new method PLSC-RP, whereby we reduced the number of voxels and retained raw SNPs. Specifically, we reduced the dimensionality of the fMRI matrix to 208 dimensions, similar to sample size, to match our simulation application above (dimensionality of the phenotype matrix was reduced to 100 dimensions according to Menon (2007), equal to *N* = 100). We compared the results of PLSC-RP to the results obtained using traditional PLSC. The findings from the original publication (Ousdal et al., 2012) served as a reference.

#### 2.8.2. PLSC-RP for high-dimensional genotypes

In Section 2.8.1, we showed that PLSC-RP is remarkably faster than PLSC when applied to high-dimensional phenotype measures. As phenotype measures, we considered brain imaging data, which is scaled metrically. In many applications, however, high-dimensional data is considered that is not continuous. To promote a wider application of PLSC-RP, we considered a second data set, containing genome-wide SNPs as genotypes together with candidate phenotype measures. SNP information statistically represents count data, since SNPs were recoded by counting the number of minor alleles per person.

To illustrate PLSC-RP for count data, we used a data set that has been published previously (Breitfeld et al., 2013). For the original study, participants were recruited from the Sorbs population. The phenotype inventory consisted, among others, of anthropometric data (weight, height, waist-to-hip ratio) and of serum vaspin (Silverman et al., 2001; Hida et al., 2005) measures extracted from blood. SNP genotyping was performed using an array-based whole-genome assay. GWA with serum vaspin was assessed by linear regression in PLINK (Purcell et al., 2007), correcting for age, gender, and BMI. The authors reported a significant association between serum vaspin concentration and six SNPs on chromosome 14, rs11160190 (*P* = 2.4 × 10^−15^), rs6575436 (*P* = 2.1 × 10^−8^), rs4905203 (*P* = 2.2 × 10^−10^), rs1956713 (*P* = 1.2 × 10^−9^), rs1956721 (*P* = 3.6 × 10^−9^) and rs11621467 (*P* = 9.2 × 10^−10^). A more detailed description of participants, phenotyping, genotyping and statistical analysis is provided in the original publication (Breitfeld et al., 2013).

In contrast to the data sets described in Section 2.8.1 (high-dimensional fMRI images), for the current data set, we considered only two experimental phenotypes (serum vaspin concentration and body height), while the genotype data contained whole-genome SNP information (359,845 SNPs for 865 individuals after quality control and removal of missing data). Hence, we applied RP to reduce the number of SNPs and kept the raw phenotype matrix. We adopted our analysis strategy from Section 2.8.1. For PLSC-RP, we reduced the dimensionality of the SNP matrix to 865 dimensions, similar to sample size. PLSC considering all 359,845 SNPs served as a reference.

#### 2.8.3. PLSC-RP for high-dimensional neuroimaging measures and high-dimensional SNPs

In Section 2.8.1 and 2.8.2, we illustrated how PLSC-RP performs when either one of the two data sets searched for association is high-dimensional. However, PLSC-RP would be of universal application if it could address both at the same time. Therefore, we generated simulation data, containing high-dimensional fMRI measures as phenotypes and high-dimensional SNPs as genotypes, following our procedure of the previous sections. We generated fMRI and SNP data of six different dimensionality combinations.

1–4 We stepwise increased the dimensionality of both the fMRI and the SNP data set, such that they contained 1000, 10,000, 20,000, or 40,000 voxels and SNPs.
5 The dimensionality of the fMRI data was higher than the dimensionality of the SNP data with 50,000 voxels and 1000 SNPs, respectively.
6 The dimensionality of the fMRI data was lower than the dimensionality of the SNP data with 1000 voxels and 50,000 SNPs, respectively.

The sample size was chosen to be 100 for both data matrices and all combinations of simulated data. Following our analysis strategy described in the previous sections, we performed PLSC on the raw data matrices, and we applied PLSC-RP to optimize runtime. We used RP to reduce the number of voxels and SNPs to 100 dimensions according to the Menon theorem (Menon, 2007) for all considered dimensionality combinations.

## 3. Results

### 3.1. PLSC-RP for high-dimensional neuroimaging measures—continuous data

First we compared the results of traditional PLSC and PLSC-RP on simulated brain imaging data of increasing dimensionality and candidate SNPs. Specifically, we compared their results with respect to the causal component, the significance of the covariance between latent variables and the runtime (Table 1). For both methods and for all simulated dimensionalities, causal voxels, and SNPs were represented in the first component, which is associated with the highest covariance, and covariances between latent variables were significant. However, PLSC and PLSC-RP differed considerably in runtime. Importantly, the higher the number of simulated voxels, the more efficient was the dimensionality reduction to 100 dimensions using RP. At maximum, runtime was reduced from 4.2 h to 35.8 s using a standard computer with usual processing power and memory (physical memory: 192 Gi, physical CPUs: two Intel Xeon E5630 CPUs with frequency 2.53GHz and 4 cores).

**Table 1:** **PLSC and PLSC-RP results for high-dimensional neuroimaging data**.

**Type of PLS analysis**	**Dimensionality of fMRI data**	**Dimensionality of SNP data**	**Causal component**	***P*-value**	**Processing time**	**Ratio**
PLSC	1000	50	1	0.0024^**^	199.325 s	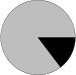
PLSC-RP	100			0.0012^**^	34.163 s		
PLSC	10,000	50	1	0.0133^*^	1738.054 s	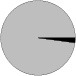
PLSC-RP	100			0.0125^*^	37.207 s		
PLSC	20,000	50	1	0.0002^***^	3958.958 s	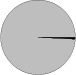
PLSC-RP	100			0.0002^***^	31.708 s		
PLSC	30,000	50	1	0.0114^*^	5949.062 s	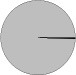
PLSC-RP	100			0.0106^*^	32.225 s		
PLSC	40,000	50	1	0.0116^*^	7595.316 s	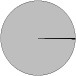
PLSC-RP	100			0.0784^*^	33.495s		
PLSC	50,000	50	1	0.0006^***^	8735.047 s	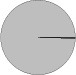
PLSC-RP	100			0.0002^***^	33.495 s		
PLSC	70,000	50	1	0.0040^**^	11671.470 s	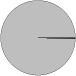
PLSC-RP	100			0.0084^**^	35.421 s		
PLSC	90,000	50	1	0.0132^*^	15112.120 s	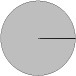
PLSC-RP	100			0.0118^*^	35.767 s		

*The dimensionality of the simulated fMRI data was increased stepwise from 1000 to 90,000 voxels. The number of SNPs was constant. The table contrasts the results of traditional PLSC (top row) and PLSC after dimensionality reduction of the fMRI data set to 100 dimensions using RP (bottom row) with regard to the component representing the causal pattern, the P-value of permutation testing for the causal component, and total processing time for performing 5000 permutations (in seconds). The last column illustrates the proportion of processing times of PLSC-RP (black) compared to traditional PLSC (gray)*.

* *P < 0.05*,

** *P < 0.01*,

*** *P < 0.001*.

**Figure 1:**
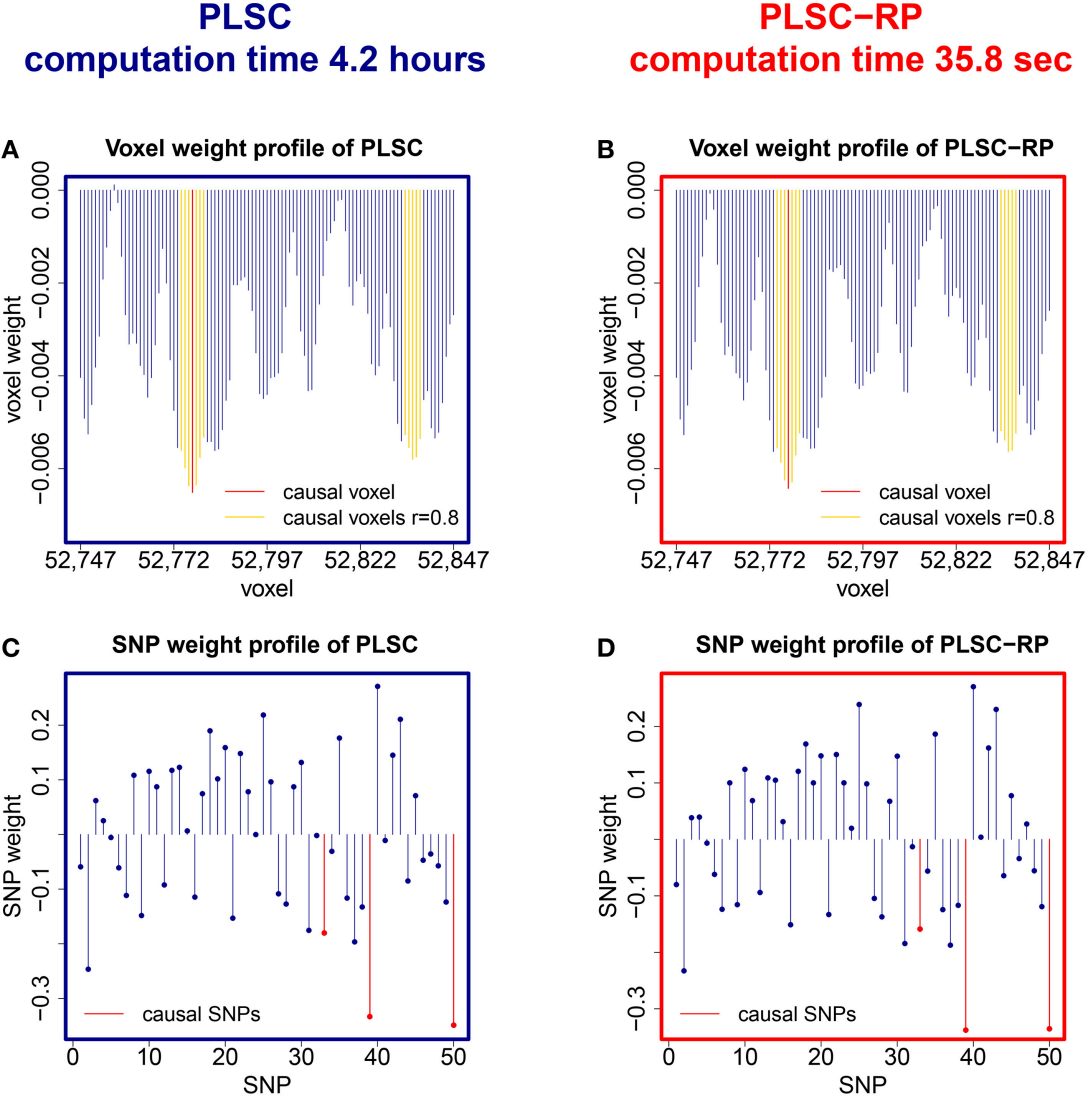
**PLSC and PLSC-RP weights for high-dimensional neuroimaging data**. **(A)** Voxel weight profile and **(C)** SNP weight profile of the causal PLSC component for 90,000 simulated voxels. **(B)** Voxel weight profile and **(D)** SNP weight profile of the causal PLSC-RP component after dimensionality reduction of the fMRI data set to 100 dimensions. PLSC and PLSC-RP provide a weight for each voxel and each SNP. The causal voxel and causal SNPs (red), and voxels in collinearity with the causal voxel (yellow), receive higher weights than non-causal voxels and SNPs (blue). For visualization purpose, the voxel weight profiles of PLSC and PLSC-RP **(A,B)** are zoomed in on a range of 100 voxels around the causal voxel.

We further compared PLSC and PLSC-RP with regard to how the individual voxels and SNPs were weighted. A sample illustration of voxel and SNP weights of PLSC for 90,000 simulated voxels and of PLSC-RP, when the dimensionality of the fMRI data set was reduced to 100 dimensions, is provided in Figure 1. Using both PLSC and PLSC-RP, causal voxels and SNPs, as highlighted in yellow and red, received the highest weights. Average weights for causal and non-causal voxels and SNPs are presented in Supplementary Table 1. It shows that voxel and SNP weights of PLSC and PLSC-RP were highly similar. This was confirmed by three similarity measures (Table 2). For all simulated dimensionalities of the fMRI data set, we observed a Pearson correlation of *s*^P^ ≈ 1, a cosine measure of *s*^C^ ≈ 1 and an extended Jaccard similarity of *s*^J^ ≈ 1. In addition, the intercepts of the ANOVA models were approximately equal to 0 and the slopes were approximately equal to 1 (Table 2). All *P*-values were smaller than 2 · 10^−16^.

**Table 2:** **Similarity of PLSC and PLSC-RP weights for high-dimensional neuroimaging data**.

**Dimensionality**	**Similarity coefficients for fMRI data**			**ANOVA for fMRI data**		**Similarity coefficients for SNP data**			**ANOVA for SNP data**	
	**Pearson's *r***	**Cosine**	**Ext. Jaccard**	**Intercept**	**Slope**	**Pearson's *r***	**Cosine**	**Ext. Jaccard**	**Intercept**	**Slope**
1000	0.9998	0.9998	0.9997	0.0007	0.9823	0.9979	0.9978	0.9957	−0.0015	0.9980
10,000	0.9966	0.9979	0.9958	−0.0002	1.0078	0.9883	0.9887	0.9777	0.0023	0.9853
20,000	0.9935	0.9961	0.9922	−0.0001	0.9834	0.9805	0.9806	0.9620	0.0023	0.9829
30,000	0.9948	0.9972	0.9944	−0.0001	0.9822	0.9819	0.9824	0.9654	−0.0006	0.9817
40,000	0.9869	0.9912	0.9826	−0.0004	0.9409	0.9727	0.9723	0.9460	0.0047	0.9754
50,000	0.9982	0.9993	0.9986	−0.0001	0.9797	0.9906	0.9908	0.9818	0.0022	0.9940
70,000	0.9800	0.9935	0.9872	−0.0003	1.0707	0.9741	0.9743	0.9499	−0.0038	0.9791
90,000	0.9974	0.9990	0.9980	−9.11 · 10^−5^	0.9768	0.9914	0.9910	0.9821	−0.0041	0.9906

*We evaluated the performance of PLSC-RP by comparing its weight profiles to the weight profiles of PLSC. We applied three similarity measures, Pearson correlation, the cosine measure and the extended Jaccard similarity. In addition, we used ANOVA for comparison. The ANOVA model represents a perfect linear relationship between the weights of PLSC and PLSC-RP, in case the intercept equals 0 and the slope equals 1*.

**Figure 2:**
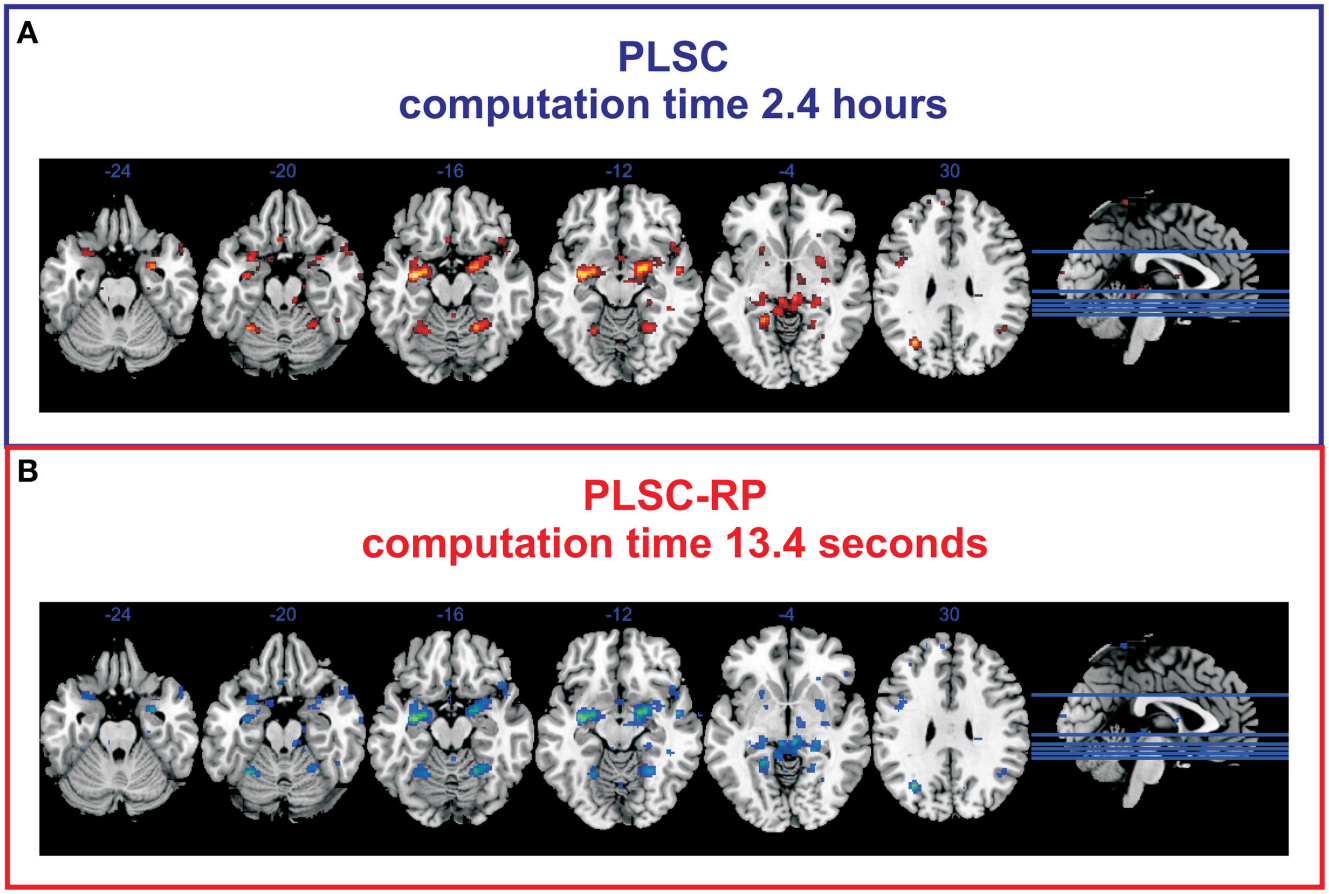
**Voxels associated to the SNPs rs10014254, rs11722038, and rs17529323 during fMRI face-matching task**. **(A)** Voxel weight profile of PLSC considering whole-brain. **(B)** Voxel weight profile of PLSC-RP after dimensionality reduction of the fMRI data set to 208 dimensions. Only voxels with top 50% weights are shown. We found an association with fMRI activity in bilateral amygdala (*x* = −22, *y* = −4, *z* = −12; *x* = 18, *y* = −4, *z* = −12), bilateral cerebellum (*x* = −28, *y* = −54, *z* = −20; *x* = 22, *y* = −54, *z* = −16), left hippocampus (*x* = −32, *y* = −10, *z* = −14), left lingual gyrus (*x* = −20, *y* = −46, *z* = −4), right putamen (*x* = 28, *y* = 4, *z* = −2), and left lateral occipital cortex (*x* = −30, *y* = −66, *z* = 28).

To verify our findings on simulated data, we compared the results of traditional PLSC and PLSC-RP regarding experimental brain imaging and genetics data. For both PLSC and PLSC-RP, we considered only SNP and voxel weights of the first component, since it already explained a large proportion of variance (72.76 and 69.08% for PLSC and PLSC-RP, respectively). The covariance of latent variables was significant by permutation testing (*P* = 0.0248 and *P* = 0.0490 for PLSC and PLSC-RP, respectively). We found that using both, PLSC and PLSC-RP, exactly the same three SNPs (rs10014254, rs11722038 and rs17529323) as reported in the original publication (Ousdal et al., 2012) were reliable. An illustration of voxels related to these SNPs is provided in Figure 2. We were able to replicate an association with amygdala activity in both hemispheres (Ousdal et al., 2012). Importantly, we found additional brain regions including cerebellum, left hippocampus, left lingual gyrus, right putamen, and left lateral occipital cortex. Voxel weight profiles of PLSC and PLSC-RP were highly similar (Supplementary Table 2). This was confirmed by three similarity measures (Pearson correlation *s*^P^ = 0.9991, cosine measure *s*^C^ = 0.9992 and Jaccard similarity *s*^J^ = 0.9985) and ANOVA (intercept = 2.14 · 10^−5^, slope = 0.9948 and *P* < 2 · 10^−16^). PLSC-RP was, however, remarkably faster than PLSC, reducing runtime from 2.4 h to 13.4 s.

### 3.2. PLSC-RP for high-dimensional SNPs—count data

Next we illustrated the performance of PLSC-RP for association analysis of a data set containing genome-wide SNPs as genotypes together with candidate phenotype measures. In contrast to the brain imaging data in Section 3.1, which was scaled metrically, SNP information statistically represents count data, since SNPs were recoded by counting the number of minor alleles per person.

We observed that both PLSC and PLSC-RP revealed a two component solution. In the first component of the phenotype weight profile, serum vaspin level was highly weighted (|***w***_vaspin_| = 0.7068 for both PLSC and PLSC-RP). Body height was most contributing to the second component (|***w***_height_| = 0.9994 for PLSC and |***w***_height_| = 0.9996 for PLSC-RP). Both components were significant by permutation testing (Edgington, 1980) ($P_{\text{vaspin}}<2.2\times 10^{-16}$ and *P*_height_ = 0.02 for both PLSC and PLSC-RP). Since the out-of-sample covariance (Le Floch et al., 2012) was much higher for the first component (*cov*_vaspin_ = 6.69 and *cov*_height_ = 0.89), we restricted our interpretation to associations with serum vaspin.The overall runtime for PLSC was 36.4 h. PLSC-RP reduced it to 4.8 min.

**Figure 3:**
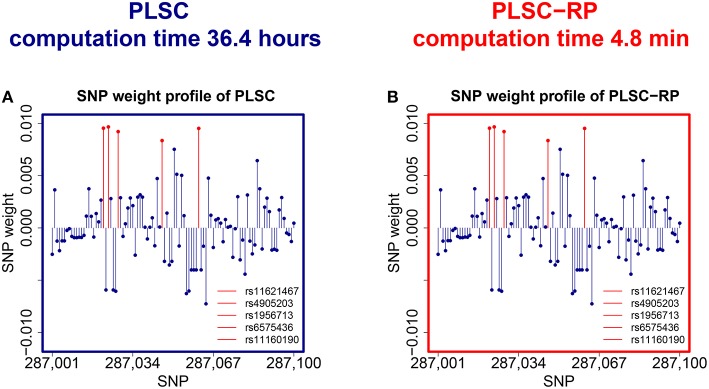
**SNPs associated with serum vaspin concentration in the Sorbs**. **(A)** SNP weight profile of PLSC applied to the original data set consisting of 359,845 SNPs. **(B)** SNP weight profile of PLSC-RP after dimensionality reduction of the SNP data set to 865 dimensions. Reliable SNPs according to bootstrapping are plotted in red. For visualization purpose, the SNP weight profiles are zoomed in on a range representing causal variants.

An illustration of the SNPs, which were associated with serum vaspin concentrations in the Sorbs, is provided in Figure 3. Using bootstrapping (Tibshirani and Efron, 1993), we showed that exactly the same SNPs on chromosome 14 that were reported in the original publication (Breitfeld et al., 2013) were reliable, including rs11160190, rs6575436, rs4905203, rs1956713, and rs11621467. We did not find an association for rs1956721 because we had to exclude this SNP due to its low call rate. The SNP weight profiles of PLSC and PLSC-RP were highly similar (Pearson correlation *s*^P^ = 0.9999, cosine measure *s*^C^ = 0.9999, Jaccard similarity *s*^J^ = 0.9999, ANOVA intercept = −1.95 · 10^−8^, slope = 0.9999993 and *P* < 2 · 10^−16^, Supplementary Table 3).

### 3.3. PLSC-RP for high-dimensional neuroimaging measures and high-dimensional SNPs—continuous and count data

According to our results presented in Section 3.1 and 3.2, PLSC-RP provides statistically the same results as PLSC when we perform dimensionality reduction of either one of the two data sets searched for association. However, PLSC-RP would be of universal application if it could address both at the same time. Therefore, we compared the results of PLSC-RP to the results obtained using traditional PLSC for simulation data containing high-dimensional fMRI measures and high-dimensional SNPs.

As for our simulation results on high-dimensional neuroimaging data (Section 3.1), causal voxels and SNPs were represented in the first component for all dimensionality combinations and for both PLSC and PLSC-RP (Table 3). The covariances between latent variables were non-significant, since chance correlations have a considerable effect in multivariate models such as PLSC if variable numbers in both data sets are excessively high compared to sample size. In terms of runtime, PLSC-RP was remarkably faster than traditional PLSC. At maximum, runtime was reduced from ca. 3757 h to 1.2 min.

**Table 3:** **PLSC and PLSC-RP results for high-dimensional neuroimaging and high-dimensional SNP data**.

**Type of PLS analysis**	**Dimensionality of fMRI data**	**Dimensionality of SNP data**	**Causal component**	***P*-value**	**Processing time**	**Ratio**
PLSC	1000	1000	1	0.2116	5.3 h	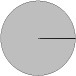
PLSC-RP	100	100		0.1440	1.11 min		
PLSC	10,000	10,000	1	0.3182	652.6 h	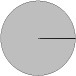
PLSC-RP	100	100		0.4582	1.07 min		
PLSC	20,000	20,000	1	0.4554	1184.7 h	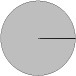
PLSC-RP	100	100		0.5322	1.15 min		
PLSC	40,000	40,000	1	0.333	3756.6 h	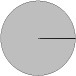
PLSC-RP	100	100		0.5184	1.17 min		
PLSC	50,000	1000	1	0.1951	149.2 h	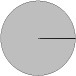
PLSC-RP	100	100		0.2344	1.28 min		
PLSC	1000	50,000	1	0.3358	184.8 h	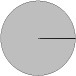
PLSC-RP	100	100		0.1586	1.09 min		

We generated simulated fMRI and SNP data of six different dimensionality combinations. The table contrasts the results of traditional PLSC on the raw data (top row) and PLSC-RP for reduced voxel and SNP data sets of 100 dimensions, respectively (bottom row). Results are compared with regard to the component representing the causal pattern, the P-value of permutation testing for the causal component, and total processing time for performing 5000 permutations (1000 permutations for 10,000 simulated voxel and SNPs, 250 permutations for 20,000 simulated voxels and SNPs, and 100 permutations for 40,000 simulated voxels and SNPs due to runtime). The last column illustrates the proportion of processing times of PLSC-RP (black) compared to traditional PLSC (gray). ^*^P < 0.05, ^**^P < 0.01, ^***^P < 0.001.

**Figure 4:**
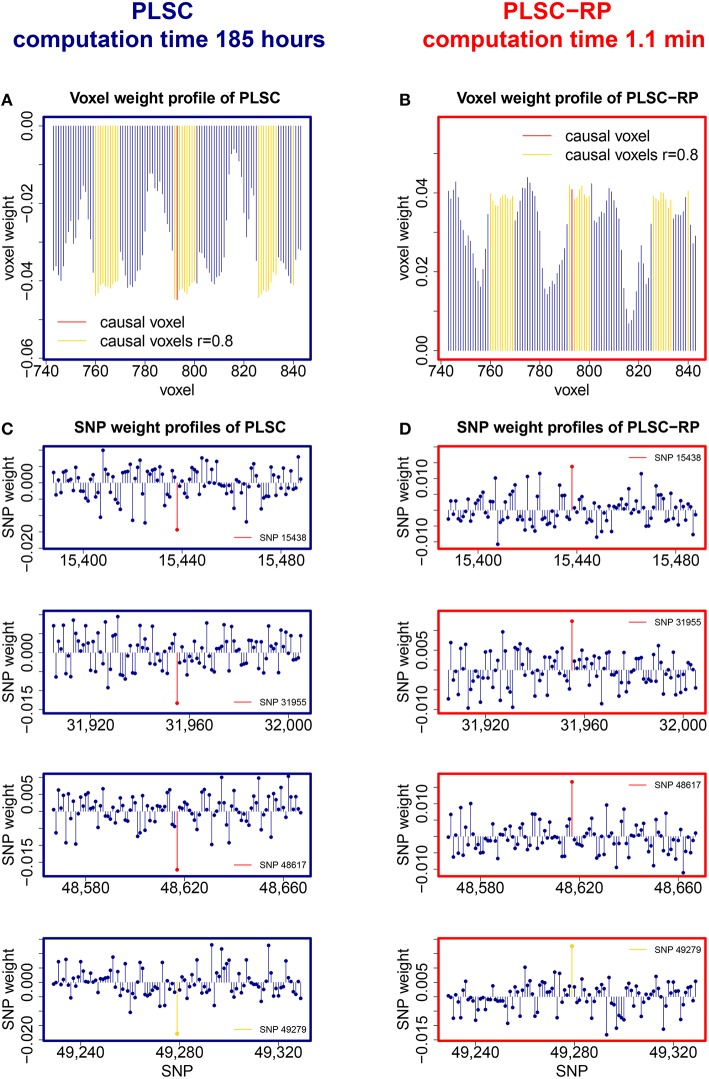
**PLSC and PLSC-RP weights for high-dimensional neuroimaging and high-dimensional SNP data**. **(A)** Voxel weight profile and **(C)** SNP weight profiles of the causal PLSC component for 1000 simulated voxels and 50,000 simulated SNPs. **(B)** Voxel weight profile and **(D)** SNP weight profiles of the causal PLSC-RP component after dimensionality reduction of both data sets to 100 dimensions. The causal voxel and causal SNPs (red), and voxels in collinearity with the causal voxel (yellow), receive higher weights than non-causal voxels and SNPs (blue). In addition to the causal SNPs, several other SNPs were found to be highly weighted, such as SNP 49,279 (bottom row, highlighted in yellow). For visualization purpose, the voxel weight profiles **(A,B)** and the SNP weight profiles **(C,D)** are zoomed in on a range representing causal variants.

Sample illustrations of voxel and SNP weights for 1000 voxels and 50,000 SNPs (dimensionality combination 6) are provided in Figure 4. In general, using both PLSC and PLSC-RP, causal voxels and SNPs got the highest weights. Weight profiles of PLSC and PLSC-RP were comparably similar (Supplementary Table 4 and Table 4). However, compared to our applications of RP for dimensionality reduction of high-dimensional continuous or high-dimensional count data, the degree of similarity was reduced, on average, from 0.99 to 0.94. The two approaches, PLSC and PLSC-RP, mainly differed in terms of weights provided for non-causal voxels and SNPs, whereas the weights for causal variables were approximately equal. Note that in Figure 4 the magnitude of PLSC and PLSC-RP voxel and SNP weights is comparably similar (especially for causal variables), whereas the direction of weights is reversed. However, the direction of weights is irrelevant, as long as the sign of both voxel and SNP weights is reversed for PLSC-RP compared to PLSC.

**Table 4:** **Comparison of PLSC and PLSC-RP weights for high-dimensional neuroimaging and high-dimensional SNP data**.

**Dimensionality**	**Dimensionality**	**Similarity coefficients for fMRI data**			**ANOVA for fMRI data**		**Similarity coefficients for SNP data**			**ANOVA for SNP data**	
**of fMRI data**	**of SNP data**	**Pearson's *r***	**Cosine**	**Ext. Jaccard**	**Intercept**	**Slope**	**Pearson's *r***	**Cosine**	**Ext. Jaccard**	**Intercept**	**Slope**
1000	1000	0.9378	0.9890	0.9782	0.0039	0.8771	0.9886	0.9886	0.9774	−0.0002	0.9887
10,000	10,000	0.9691	0.9829	0.9664	0.0008	0.9260	0.9845	0.9845	0.9695	3.61 · 10^−5^	0.9846
20,000	20,000	0.8816	0.9150	0.8432	−0.0019	0.7638	0.9402	0.9402	0.8872	−4.58 · 10^−5^	0.9402
40,000	40,000	0.8252	0.8945	0.8092	0.0004	0.8373	0.9306	0.9307	0.8704	−5.77 · 10^−6^	0.9306
50,000	1000	0.8865	0.9618	0.9265	−0.0012	0.7378	0.9522	0.9928	0.9857	0.0004	0.9525
1000	50,000	0.9554	0.9928	0.9857	−0.0001	0.9888	0.9895	0.9895	0.9793	−1.03 · 10^−5^	0.9895

*The table summarizes the results of the comparison of PLSC and PLSC-RP weight profiles. We applied three different similarity measures, Pearson correlation, the cosine measure, and the extended Jaccard similarity. In addition, we used ANOVA for comparison*.

In addition to our causal SNPs highlighted in Figure 4, several other SNPs were provided with high weights by PLSC and PLSC-RP, including e.g., SNP 14,891, SNP 20,330, SNP 22,630, SNP 23,349, and SNP 49,279 in the case of dimensionality combination 6. All of these SNPs were actually linked to the causal voxel, as revealed by means of Pearson correlation (*r*_SNP 14, 891_ = −0.3857, *r*_SNP 20, 330_ = −0.3471, *r*_SNP 22, 630_ = −0.3230, *r*_SNP 23, 349_ = −0.3293 and *r*_SNP 49, 279_ = 0.3825).

### 3.4. Comparison of PLSC-RP with and without gram–schmidt orthogonalization

For dimensionality reduction using RP, random matrices need to be orthogonalized in order to preserve distances between the original points in the low-dimensional space. Unfortunately, Gram–Schmidt orthogonalization is computationally expensive. However, it has been shown by Hecht-Nielsen (1994) that in high-dimensional spaces, there already exists a large number of nearly orthogonal vectors, such that high-dimensional random matrices are sufficiently close to orthogonal and orthogonalization using the Gram–Schmidt algorithm can be omitted. In order to quantify whether Gram–Schmidt orthogonalization is necessary in high-dimensional genetic neuroimaging data sets, we reran the analyses from Section 3.1 once using the Gram–Schmidt algorithm to orthogonalize random matrices and once without orthogonalization. Random matrices for the analyses with and without Gram–Schmidt orthogonalization were the same but differed from the random matrices in Section 3.1. We compared

1. the weights of traditional PLSC and of PLSC-RP using random matrices that were orthogonalized using Gram–Schmidt algorithm,
2. the weights of traditional PLSC and of PLSC-RP when we omitted Gram–Schmidt orthogonalization,
3. the weights of PLSC-RP using random matrices with and without Gram–Schmidt orthogonalization,

using the similarity measures Pearson correlation, cosine measure and extended Jaccard similarity as well as ANOVA. Results are illustrated in Table 5. It shows that the similarity between the weights of traditional PLSC and PLSC-RP using Gram–Schmidt algorithm for orthogonalization was only slightly higher as (for 1000, 10,000, and 40,000 voxels) or close to identical to (for 20,000, 30,000, 50,000, 70,000, and 90,000 voxels) the similarity between the weights of traditional PLSC and PLSC-RP when we omitted Gram–Schmidt orthogonalization. Furthermore, the similarity between the weights of PLSC-RP with and without Gram–Schmidt orthogonalization was higher than the similarity between the weights of either method and traditional PLSC. More specifically, the higher the dimensionality of the simulated fMRI data and therefore the higher the number of rows of the random matrices, the higher was the similarity between the weights of PLSC-RP with and without Gram–Schmidt orthogonalization (e.g., Pearson correlation for 1000 voxels $s_{\text{MRI}}^{\text{P}}=0.9998$ and $s_{\text{SNP}}^{\text{P}}=0.9923$, Pearson correlation for 10,000 voxels $s_{\text{MRI}}^{\text{P}}=0.9998$ and $s_{\text{SNP}}^{\text{P}}=0.9996$, Pearson correlation for 20,000 voxels or more $s_{\text{MRI}}^{\text{P}}=0.9999$ and $s_{\text{SNP}}^{\text{P}}=0.9999$).

**Table 5:** **Similarity of weights of traditional PLSC and of PLSC-RP using random matrices with and without Gram–Schmidt orthogonalization**.

**Dim.**	**Comp.**	**Similarity coefficients for fMRI data**			**ANOVA for fMRI data**		**Similarity coefficients for SNP data**			**ANOVA for SNP data**	
		**Pearson's *r***	**Cosine**	**Ext. Jaccard**	**Intercept**	**Slope**	**Pearson's *r***	**Cosine**	**Ext. Jaccard**	**Intercept**	**Slope**
1000	A	0.9954	0.9977	0.9954	0.0019	0.9505	0.9891	0.9890	0.9783	0.0012	0.9891
1000	B	0.9940	0.9969	0.9939	0.0022	0.9421	0.9861	0.9861	0.9725	0.0009	0.9861
1000	C	0.9998	0.9999	0.9998	−0.0003	0.9923	0.9994	0.9994	0.9988	0.0003	0.9994
10,000	A	0.9981	0.9987	0.9975	−0.0002	1.0147	0.9919	0.9920	0.9841	0.0034	0.9872
10,000	B	0.9976	0.9983	0.9966	−0.0003	1.0201	0.9905	0.9905	0.9812	0.0044	0.9846
10,000	C	0.9998	0.9998	0.9997	0.0001	1.0056	0.9996	0.9996	0.9992	−0.0009	0.9984
20,000	A	0.9965	0.9979	0.9959	6.22 · 10^−5^	1.0037	0.9914	0.9915	0.9832	−0.0008	0.9909
20,000	B	0.9967	0.9981	0.9961	4.55 · 10^−5^	1.0023	0.9918	0.9919	0.9840	−0.0011	0.9910
20,000	C	0.9999	0.9999	0.9999	−1.74 · 10^−5^	0.9984	0.9999	0.9999	0.9999	−0.0003	0.9997
30,000	A	0.9932	0.9972	0.9944	−1.32 · 10^−4^	0.9807	0.9800	0.9806	0.9619	0.0002	0.9809
30,000	B	0.9930	0.9972	0.9944	−1.39 · 10^−4^	0.9799	0.9794	0.9800	0.9608	0.0004	0.9805
30,000	C	0.9999	0.9999	0.9999	5.60 · 10^−6^	0.9993	0.9999	0.9999	0.9999	−0.0002	1.0002
40,000	A	0.9855	0.9896	0.9793	−4.75 · 10^−4^	0.9295	0.9666	0.9667	0.9356	−7.68 · 10^−4^	0.9665
40,000	B	0.9860	0.9900	0.9802	−4.63 · 10^−4^	0.9313	0.9682	0.9683	0.9385	−8.41 · 10^−4^	0.9680
40,000	C	0.9999	0.9999	0.9999	1.03 · 10^−5^	1.0013	0.9999	0.9999	0.9998	−8.85 · 10^−5^	0.9999
50,000	A	0.9942	0.9977	0.9955	−2.17 · 10^−4^	0.9584	0.9831	0.9821	0.9648	−0.0078	0.9750
50,000	B	0.9940	0.9977	0.9954	−2.24 · 10^−4^	0.9571	0.9826	0.9816	0.9639	−0.0080	0.9744
50,000	C	0.9999	0.9999	0.9999	6.70 · 10^−6^	0.9988	0.9999	0.9999	0.9999	0.0001	0.9998
70,000	A	0.9944	0.9984	0.9967	9.79 · 10^−5^	0.9765	0.9896	0.9894	0.9791	−0.0039	0.9943
70,000	B	0.9943	0.9983	0.9967	9.47 · 10^−5^	0.9772	0.9894	0.9892	0.9787	−0.0040	0.9943
70,000	C	0.9999	0.9999	0.9999	3.67 · 10^−6^	1.0008	0.9999	0.9999	0.9999	0.0002	1.0002
90,000	A	0.9935	0.9977	0.9955	−1.07 · 10^−4^	0.9718	0.9858	0.9858	0.9720	−8.68 · 10^−4^	0.9856
90,000	B	0.9934	0.9977	0.9954	−1.07 · 10^−4^	0.9716	0.9856	0.9856	0.9716	−9.41 · 10^−4^	0.9853
90,000	C	0.9999	0.9999	0.9999	−4.33 · 10^−7^	0.9999	0.9999	0.9999	0.9999	−7.29 · 10^−5^	0.9999

*We compared the weights of traditional PLSC and of PLSC-RP with and without Gram–Schmidt orthogonalization using three similarity measures, Pearson correlation, the cosine measure and the extended Jaccard similarity. Furthermore, we applied ANOVA for comparison. (A) Comparison of weights of traditional PLSC and of PLSC-RP using random matrices that were orthogonalized using Gram–Schmidt algorithm. (B) Comparison of weights of traditional PLSC and of PLSC-RP when we omitted Gram–Schmidt orthogonalization. (C) Comparison of weights of PLSC-RP using random matrices with and without Gram–Schmidt orthogonalization*.

### 3.5. Variability of PLSC-RP results

Since for PLSC-RP dimensionality is reduced using RP, the results will vary slightly in every run of the algorithm due to the random draw of the random matrices ***R*** in Equation (4) or ***R***_***1***_ and ***R***_***2***_ in Equation (8). In order to quantify this variability, we reran the analyses from the previous sections 10 times each using different random matrices in every run of PLSC-RP. The results are illustrated in Table 6. It shows that the variability of PLSC-RP weights was very small in general. For simulated fMRI data of 90,000 voxels and 50 candidate SNPs (Figure 1), it averaged 7.93 · 10^−8^±5.58 · 10^−8^ for voxel weights and 7.77 · 10^−4^±3.84 · 10^−4^ for SNP weights. For simulated high-dimensional fMRI and high-dimensional SNP data (Figure 4), it accounted for, on average, 5.05 · 10^−5^±3.27 · 10^−5^ for voxel weights and 1.13 · 10^−6^±9.19 · 10^−7^ for SNP weights. For experimental genotype-phenotype data, the variability of PLSC-RP results was even smaller. In the fMRI face-matching task (Figure 2), it averaged 4.10 · 10^−9^±5.15 · 10^−9^ for voxel weights and 7.38 · 10^−4^±1.09 · 10^−3^ for SNP weights. However, the variability of the causal SNPs rs10014254, rs11722038 and rs17529323 was very low with 5.92 · 10^−7^±1.72 · 10^−9^ on average. The variability of non-causal SNPs was considerably higher and accounted for, on average, 2.41 · 10^−3^±8.03 · 10^−4^. For the SNPs associated with serum vaspin concentration in the Sorbs (Figure 3), we determined similar variability results. For phenotype weights, we determined an average variance of 3.69 · 10^−4^±4.63 · 10^−4^ and for SNP weights 1.29 · 10^−9^±1.84 · 10^−9^. The variability of the causal SNPs rs11621467, rs4905203, rs1956713, rs6575436, and rs11160190 was even smaller with 1.02 · 10^−9^±6.73 · 10^−10^ on average.

**Table 6:** **Variability of PLSC-RP phenotype and genotype weights**.

**High-dimensional neuroimaging data: voxel weight variance** = 7.93 · 10^**−8**^±5.58 · 10^**−8**^, **SNP weight variance** = 7.77 · 10^**−4**^±3.84 · 10^**−4**^				
	**Voxel-No./SNP-No**.	**Voxel weight/SNP weight**	**Variance of weights**	**Average percentage deviation of weights(%)**
Low	Voxel 69,627	−0.0053647	2.42 · 10^−9^	4.02
⋮	Voxel 52,777 (causal)	−0.0064222	6.45 · 10^−8^	4.41
High	Voxel 2481	0.0001969	5.11 · 10^−7^	72.22
Low	SNP 8	0.1002331	1.77 · 10^−4^	5.16
⋮	Causal SNPs	−0.2771739	9.72 · 10^−4^±4.51 · 10^−4^	6.52
High	SNP 24	0.0196767	1.95 · 10^−3^	37.52
**Functional MRI face-matching task: voxel weight variance** = 4.10 · 10^−9^±5.15 · 10^−9^, **SNP weight variance** = 7.38 · 10^−4^±1.09 · 10^−3^				
	**Voxel-No./SNP-No**.	**Voxel weight/SNP weight**	**Variance of weights**	**Average percentage deviation of weights(%)**
Low	Voxel 77,179	−0.0010709	1.17 · 10^−13^	0.04
⋮	Causal voxels	−0.0059789	2.85 · 10^−9^±2.67 · 10^−9^	0.50
High	Voxel 98,449	−0.0006442	8.75 · 10^−8^	28.80
Low	SNP 1, 2, and 3 (causal)	−0.5757151	5.92 · 10^−7^±1.72 · 10^−9^	0.15
High	SNP 4 and 5	0.0426746	2.41 · 10^−3^±8.03 · 10^−4^	12.14
**Serum vaspin concentration: phenotype weight variance** = 3.69 · 10^−4^±4.63 · 10^−4^, **SNP weight variance** = 1.29 · 10^−9^±1.84 · 10^−9^				
	**Phenotype/SNP-No**.	**Phenotype weight/SNP weight**	**Variance of weights**	**Average percentage deviation of weights(%)**
Low	Vaspin	0.7067676	1.02 · 10^−4^	0.87
High	Height	0.0294468	9.04 · 10^−4^	10.27
Low	rs16960334	−0.0002319	1.82 · 10^−15^	0.02
⋮	Causal SNPs	0.0092556	1.02 · 10^−9^±6.73 · 10^−10^	0.18
High	rs16824418	−0.0004946	7.51 · 10^−8^	5.59
**High-dimensional neuroimaging and SNP data: voxel weight variance** = 5.05 · 10^−5^±3.27 · 10^−5^**, SNP weight variance** = 1.13 · 10^−6^±9.19 · 10^−7^				
	**Voxel-No./SNP-No**.	**Voxel weight/SNP weight**	**Variance of weights**	**Average percentage deviation of weights(%)**
Low	Voxel 792	0.0419922	3.89 · 10^−6^	4.80
⋮	Voxel 793 (causal)	0.0408320	1.22 · 10^−5^	11.09
High	Voxel 516	0.0003213	1.70 · 10^−4^	78.17
Low	SNP 10,840	0.0045882	2.76 · 10^−8^	4.33
⋮	Causal SNPs	0.0142486	6.49 · 10^−7^±2.33 · 10^−7^	4.62
High	SNP 49,164	0.0006524	1.08 · 10^−5^	88.81

*We assessed the variability of PLSC-RP phenotype and genotype weights by performing 10 runs of PLSC-RP with different choices of random matrices on the same data sets. The table illustrates the exact weight, the variability and the average percentage deviation of weights for selected phenotype variables and SNPs, respectively. Weights and variability estimates of phenotypes and SNPs with minimum variability are shown in the top row, followed by weights and variability estimates of causal phenotypes and SNPs, and finally weights and variability estimates of phenotypes and SNPs with maximum variability*.

## 4. Discussion

### 4.1. Accuracy of PLSC-RP results depending on the number of original variables

Here we report a new method for efficiently performing multivariate analysis of high-dimensional genotype-phenotype association data, which we termed PLSC-RP. In a simulation series containing high-dimensional brain imaging measures of increasing voxel numbers as phenotypes and candidate SNPs as genotypes, we compared PLSC-RP to traditional PLSC and demonstrated that they provide statistically highly similar results, independent of the number of simulated voxels. Importantly, the higher the dimensionality, the more the processing time was reduced using PLSC-RP instead of PLSC.

PLSC-RP is independent of the original dimensionality, because dimensionality reduction is performed according to the Johnson-Lindenstrauss lemma (Johnson and Lindenstrauss, 1984). The Johnson-Lindenstrauss lemma states that if we pick a random subspace with reduced dimension *k* of an originally high-dimensional data, the pairwise distances between the original data points are preserved relative to an error ϵ, which we are willing to accept. Thereby, the reduced dimension *k* is logarithmic in the sample size *N*, which implies that random projections, and thus PLSC-RP, are independent of the original dimensionality. Since a reasonably high sample size *N* is sufficient to ensure the accuracy of results, regardless of the number of original variables, PLSC-RP is optimal in large scale settings. This includes genetic neuroimaging studies, where the high number of variants in the human genome and the high number of voxels in the brain complicate the identification of variations that are causally related to a particular disease (Schork et al., 2007).

In order to verify our simulation results, we applied traditional PLSC and PLSC-RP to an experimental genetic neuroimaging data set that has been published previously (Ousdal et al., 2012). We were able to replicate literature findings using both PLSC and PLSC-RP, with PLSC-RP being again significantly faster than PLSC. Specifically, we found three SNPs in high LD, rs10014254, rs11722038, and rs17529323, to be significantly associated to amygdala activity. These SNPs are located upstream of the Paired-like homeobox 2b (PHOX2B) gene, which is known to regulate the expression of enzymes necessary for the biosynthesis of monoamines (Brunet and Pattyn, 2002). Thus, we verified the authors hypothesis (Ousdal et al., 2012) that the monoaminergic signaling pathway plays a central role in the regulation of amygdala activity. In contrast to Ousdal et al. (2012), who only considered the amygdala peak voxels to search for influencing SNPs, we considered whole brain measures. Therefore, we also found some other brain regions to be involved during the emotional face-matching task, including cerebellum, left hippocampus, left lingual gyrus and right putamen. All these brain regions have been shown to be increasingly activated during processing of negative emotional faces (Fusar-Poli et al., 2009; Surguladze et al., 2010; Benedetti et al., 2011; Schraa-Tam et al., 2012; Demenescu et al., 2013). To date, it is not confirmed by the literature that individual differences in activation of these brain regions might be explained by variation of PHOX2B SNPs. However, we found evidence for an association of hippocampus with another gene influencing the monoaminergic signaling pathway, the gene that encodes the enzyme MAOA (Lee and Ham, 2008).

### 4.2. Accuracy of PLSC-RP results depending on the data type

We compared PLSC-RP to traditional PLSC on a data set containing genome-wide SNPs as genotypes together with candidate phenotype measures (body height and serum vaspin concentration extracted from blood, specifically). In contrast to the brain imaging measures, which we selected as high-dimensional phenotypes in the first application and which were scaled metrically, SNP information statistically represents count data, since PLSC was performed under the assumption of an additive genetic model. We showed that PLSC-RP provides statistically equivalent results to PLSC, despite significant savings in runtime. Therefore, dimensionality reduction using RP is data type independent. Using both PLSC and PLSC-RP, we were further able to replicate literature findings (Breitfeld et al., 2013). In the original publication, six SNPs on chromosome 14, mapping between serpinA1 and serpinA4, were shown to be significantly associated to serum vaspin measures. With the exception of one SNP that we had to exclude due to its low call rate, we verified these findings. We did not find any additional SNPs, since the authors in the original publication (Breitfeld et al., 2013) already performed a genome-wide screening.

### 4.3. Accuracy of PLSC-RP results for both high-dimensional neuroimaging and SNP data

Finally, we applied PLSC-RP on a simulated data set containing both high-dimensional brain imaging and high-dimensional SNP measures of different dimensionality combinations. We showed that PLSC-RP was able to detect causal voxels and SNPs with high accuracy, despite slightly reduced similarity to the results provided by PLSC compared to the applications of PLSC-RP and PLSC for either high-dimensional neuroimaging measures or high-dimensional SNPs. Again, PLSC-RP was significantly faster than PLSC. At maximum, runtime was reduced from 22 weeks to 1.2 min. Thus, we strongly recommend the use of PLSC-RP even if both data sets are high-dimensional.

### 4.4. Comparison of PLSC-RP with and without gram–schmidt orthogonalization

For dimensionality reduction using RP, random matrices need to be orthogonal. To quantify whether Gram–Schmidt orthogonalization is necessary or whether high-dimensional random matrices are sufficiently close to orthogonal without orthogonalization, we repeated our analyses from Section 3.1 once using the Gram–Schmidt algorithm to orthogonalize random matrices and once without orthogonalization. We showed that the weights of PLSC-RP using Gram–Schmidt algorithm and the weights of PLSC-RP when we omitted orthogonalization were close to identical for all simulated fMRI data sets. Thus, the quality of PLSC-RP results is not depended on the Gram-Schmidt algorithm in high-dimensional association analyses, such that a preceding orthogonalization of random matrices can be safely omitted. This is also suggested, since the Gram-Schmidt algorithm is computationally expensive. We further observed that the similarity was slightly higher, the higher the dimensionality of the simulated fMRI data. Therefore, an orthogonalization becomes less necessary, the more voxels and/or SNPs are considered. In genetic neuroimaging, where the data sets usually capture the whole brain or the whole genome, respectively, the Gram-Schmidt algorithm can be omitted. However, for smaller data sets, we recommend to orthogonalize random matrices.

### 4.5. Variability of PLSC-RP results

Since the dimensionality reduction in PLSC-RP depends on the choice of random matrices, the algorithm produces slightly different results in every run. To quantify this variability, we repeated our analyses 10 times each, using different random matrices in every run of PLSC-RP. We showed that the variability of PLSC-RP results was very small in general. The highest variability we determined for a single genotypic or phenotypic variable was 0.0025. On average, however, variability was much lower. Thus, PLSC-RP is appropriate for both exploratory analyses, in order to detect causal SNPs and phenotypes maximizing the joint covariance in an association study, and replication analyses, when multiple runs of the algorithm are performed. Repeatability of PLSC-RP results is assured even if different random matrices are selected for dimensionality reduction in every run of the algorithm.

We further observed that variability of PLSC-RP results was smaller the higher the sample size of our data sets. For our simulation experiments, the sample size was chosen to be 100, and the variability of PLSC-RP weights including all voxels and SNPs was between 1.95 · 10^−3^ and 2.42 · 10^−9^. In contrast, when we used PLSC-RP to search for genome-wide SNPs associated with serum vaspin concentration, sample size was equal to 865, and the variability of PLSC-RP weights including the phenotypes serum vaspin concentration and body height as well as all SNPs was between 9.04 · 10^−4^ and 1.82 · 10^−15^. Dimensionality reduction in PLSC-RP depends on the sample size *N*, and, according to the Johnson-Lindenstrauss lemma, it assures to preserve the distances between the original data points when *N* is logarithmic in the reduced dimension *k* (Johnson and Lindenstrauss, 1984). We reduced the dimensionality of the fMRI and/or SNP matrices to sample size in all applications. Thus, the higher the sample size, the higher was also the accuracy of distance preservation for a given probability of success (Table 7). The higher the accuracy of the distance preservation, the higher the degree of similarity between the results of PLSC-RP and traditional PLSC, and likewise, the higher also the degree of similarity between the results of multiple runs of PLSC-RP using different random matrices.

**Table 7:** **Accuracy of the distance preservation ϵ depending on sample size ***N*** and reduced dimension ***k*** for a given probability of success 1−*N*^−β^ in PLSC-RP**.

***N***	**100**	200	**208**	500	865	1000	2000	5000	8000	10,000
*k*	100	200	208	500	865	1000	2000	5000	8000	10,000
**1**−*N*^−β^	0.95	0.95	0.95	0.95	0.95	0.95	0.95	0.95	0.95	0.95
ϵ	0.9882	0.7374	0.7251	0.4968	0.3909	0.3667	0.2698	0.1790	0.1448	0.1309

*We determined the accuracy of the distance preservation ϵ according to the Menon theorem in Equation (5) for a fixed probability of success 1−N^−β^ = 0.95 and a fixed reduced dimension k similar to sample size. Accordingly, the higher the sample size, the higher also the accuracy of distance preservation. Bold values represent sample sizes of data sets introduced in this study*.

### 4.6. PLSC-RP in comparison to other dimensionality reduction methods in genetic neuroimaging

With combining PLSC and RP, we made two major contributions to the analysis of genetic neuroimaging data. First, in genetic neuroimaging studies, the number of variables usually exceeds the number of observations, such that multivariate methods encounter critical over-fitting issues (Le Floch et al., 2012). Our new technique uses RP for dimensionality reduction in order to circumvent this problem. Previous studies (Hibar et al., 2011a,b; Le Floch et al., 2012; Hua et al., 2015) also contributed to this end by implementing univariate filters or PCA as pre-processing step. However, they performed dimensionality reduction on either the genetic or the neuroimaging data set. In contrast, to illustrate the PLSC-RP methodology, we systematically used a two-stepped approach. First, we applied PLSC-RP for multivariate analysis of data sets containing either high-dimensional neuroimaging measures or high-dimensional SNPs. Neuroimaging measures are scaled metrically, but SNPs are counts. Therefore, we demonstrated that PLSC-RP is data-type independent. Then, we considered data sets containing a combination of high-dimensional neuroimaging measures and high-dimensional SNPs, and performed dimensionality reduction on both domains. This has not been done before.

Our second and most important contribution is related to computational efficiency. Previous studies (Hibar et al., 2011a,b; Le Floch et al., 2012; Hua et al., 2015) implemented univariate filters or PCA as pre-processing step, which are computationally very expensive procedures. In contrast, PLSC-RP is able to dramatically reduce runtime and enables researchers to analyze truly high-dimensional data sets, even if there is no powerful compute server available in the lab. Hence, our study is the first of its kind that implements dimensionality reduction both to overcome critical over-fitting issues and to reduce runtime.

### 4.7. Potential applications

In this study, we applied PLSC-RP to efficiently assess genome-wide and whole-brain associations. Combining neuroimaging data with genetic information is a rapidly growing research approach, enabling the integration of information from two of the major methodological advances introduced in the past 30 years, namely sequencing of the entire human genome and fMRI in humans. However, the application of PLSC-RP is not limited to the combined analysis of genotypes and brain imaging phenotypes. It opens up a wide range of possible uses far beyond imaging genetics. PLSC-RP might be considered for fusion of several brain imaging techniques, such as MRI, positron emission tomography (PET), diffusion tensor imaging (DTI), or magnetoencephalography (MEG) in order to profit from the benefits of each modality (Sui et al., 2012). It is suitable for the integrated analysis of disease status and multiple types of “omics” data, such as genomics, epigenomics, and transcriptomics, aiming to understand signs of malfunction that cause diseases. Furthermore, it can be applied to investigate how concentrations of biomolecules in different tissues or different species, such as mice and humans, are associated to each other. To summarize, PLSC-RP is appropriate for any integrative analysis which combines information from multiple sources and has therefore a multitude of potential applications. Since PLSC-RP depends on sample size only and is independent of the number of variables, it is especially attractive for large-scale multicenter association studies or other data sharing projects.

## Author contributions

CG, SB, and AH designed the theoretical approach. CG implemented the approach. CG designed and performed the simulation experiment. AT, PK, LW, and OA collected the experimental data. CG analyzed the data. PK, AT, LW, OA, MS, and AV contributed materials. AH, JN, and SB advised on statistics. CG wrote the manuscript. JN, SB, PK, AT, LW, OA, MS, AV, and AH edited the manuscript and approved its final version.

### Conflict of interest statement

The authors declare that the research was conducted in the absence of any commercial or financial relationships that could be construed as a potential conflict of interest.

## Supplementary material

### Web resources

Software code, sample input data and complete documentation are available at http://www.o-brain-project.de/en/methoden/.

## References

[B1] 1000 Genomes Project Consortium Abecasis, G. R. Altshuler R. M. Auton A. Brooks L. D. Durbin R. M.. (2010). A map of human genome variation from population-scale sequencing. Nature 467, 1061–1073. 10.1038/nature09534 20981092 PMC3042601

[B2] Achlioptas D. (2001). Database-friendly random projections, in Proceedings of the 20th ACM SIGMOD-SIGACT-SIGART Symposium on Principles of Database Systems (Santa Barbara, CA), 274–281.

[B3] Anderberg M. R. (1973). Cluster Analysis for Applications. Probability and Mathematical Statistics. New York, NY: Academic Press.

[B4] Baker M. (2012). Functional genomics: the changes that count. Nature 482, 257–262. 10.1038/482257a 22318607

[B5] Barad M. Greicius M. D. Mackey S. (2009). Imaging the CNS correlates of neuropathic pain. Neuropathic Pain 15, 30–46. 10.1212/01.con.0000348853.20265.b7

[B6] Bellman R. E. (1957). Dynamic Programming. Princeton Landmarks in Mathematics. Princeton, NJ: Princeton University Press.

[B7] Bellman R. E. (1960). Directions of mathematical research in nonlinear circuit theory. IRE Trans. Circ. Theor. 7, 542–553.

[B8] Benedetti F. Radaelli D. Poletti S. Falini A. Cavallaro R. Dallaspezia S.. (2011). Emotional reactivity in chronic schizophrenia: structural and functional brain correlates and the influence of adverse childhood experiences. Psychol. Med. 41, 509–519. 10.1017/S0033291710001108 20529416

[B9] Bigos K. L. Weinberger D. R. (2010). Imaging genetics-days of future past. Neuroimage 53, 804–809. 10.1016/j.neuroimage.2010.01.035 20080192

[B10] Bingham E. Mannila H. (2001). Random projection in dimensionality reduction: applications to image and text data, in Proceedings of the 7th International Conference on Knowledge Discovery and Data Mining (San Francisco, CA), 245–250.

[B11] Björck A. (1967). Solving linear least squares problems by Gram-Schmidt orthogonalization. BIT 7, 1–21.

[B12] Bookstein F. L. (1994). Partial least squares: a dose-response model for measurement in the behavioral and brain sciences. Psycoloquy 5.

[B13] Breitfeld J. Tönjes A. Böttcher Y. Schleinitz D. Wiele N. Marzi C.. (2013). Genetic variation in the vaspin gene affects circulating serum vaspin concentrations. Int. J. Obes. (Lond.) 37, 861–866. 10.1038/ijo.2012.133 22907691

[B14] Brunet J. F. Pattyn A. (2002). PHOX2 genes-from patterning to connectivity. Curr. Opin. Genet. Dev. 12, 435–440. 10.1016/S0959-437X(02)00322-2 12100889

[B15] Carre J. M. Fisher P. M. Manuck S. B. Hariri A. R. (2010). Interaction between trait anxiety and trait anger predict amygdala reactivity to angry facial expressions in men but not women. Soc. Cogn. Affect. Neurosci. 7, 213–221. 10.1093/scan/nsq101 21183456 PMC3277369

[B16] Chambers J. M. Freeny A. Heiberger R. M. (1992). Statistical Models in S, chapter 5. Analysis of variance. Designed experiments. Pacific Grove, CA: Wadsworth and Brooks/Cole.

[B17] Crawford D. C. Nickerson D. A. (2005). Definition and clinical importance of haplotypes. Annu. Rev. Med. 56, 303–320. 10.1146/annurev.med.56.082103.104540 15660514

[B18] Dasgupta S. (2000). Experiments with random projection, in Proceedings of the 16th Conference on Uncertainty in Artificial Intelligence (Stanford, CA), 143–151.

[B19] Dasgupta S. Gupta A. (1999). An Elementary Proof of the Johnson-Lindenstrauss Lemma. Technical report, tr-99-006, International Computer Science Institute, Berkeley, California.

[B20] Demenescu L. R. Kortekaas R. Cremers H. R. Renken R. J. van Tol M. J. van der Wee N. J.. (2013). Amygdala activation and its functional connectivity during perception of emotional faces in social phobia and panic disorder. J. Psychiatr. Res. 47, 1024–1031. 10.1016/j.jpsychires.2013.03.020 23643103

[B21] Edgington E. S. (1980). Randomization Tests. New York, NY: Marcel Dekker, Inc.

[B22] Filippini N. Rao A. Wetten S. Gibson R. A. Borrie M. Guzman D.. (2009). Anatomically-distinct genetic associations of APOE epsilon4 allele load with regional cortical atrophy in alzheimer's disease. Neuroimage 44, 724–728. 10.1016/j.neuroimage.2008.10.003 19013250

[B23] Frankl P. Maehara H. (1988). The Johnson-Lindenstrauss lemma and the sphericity of some graphs. J. Comb. Theory B 44, 355–362.

[B24] Friston K. J. Ashburner J. Kiebel S. J. Nichols T. E. Penny W. D. (2007). Statistical Parametric Mapping: The Analysis of Functional Brain Images. Amsterdam; Boston, MA; Heidelberg; London; New York, NY; Oxford; Paris; San Diego, CA; San Francisco, CA; Singapore; Sydney, NSW; Tokyo: Academic Press.

[B25] Fusar-Poli P. Placentino A. Carletti F. Landi P. Allen P. Surguladze S.. (2009). Functional atlas of emotional faces processing: a voxel-based meta-analysis of 105 functional magnetic resonance imaging studies. J. Psychiatry Neurosci. 34, 418–432. 19949718 PMC2783433

[B26] Ge T. Schumann G. Feng J. (2013). Imaging genetics-towards discovery neuroscience. Quant. Biol. 1, 227–245. 10.1007/s40484-013-0023-1

[B27] Goel N. Bebis G. Nefian A. (2005). Face recognition experiments with random projection, in Proceedings of the SPIE Conference on Biometric Technology for Human Identification II (Orlando, FL), 426–437.

[B28] Gottesman I. I. Gould T. D. (2003). The endophenotype concept in psychiatry: etymology and strategic intentions. Am. J. Psychiatry 160, 636–645. 10.1176/appi.ajp.160.4.636 12668349

[B29] Gottesman I. I. Shields J. (1967). A polygenic theory of schizophrenia. Proc. Natl. Acad. Sci. U.S.A. 58, 199–205. 5231600 10.1073/pnas.58.1.199 PMC335617

[B30] Grellmann C. Bitzer S. Neumann J. Westlye L. T. Andreassen O. A. Villringer A.. (2015). Comparison of variants of canonical correlation analysis and partial least squares for combined analysis of MRI and genetic data. Neuroimage 107, 289–310. 10.1016/j.neuroimage.2014.12.025 25527238

[B31] Haines J. L. Hauser M. A. Schmidt S. Scott W. K. Olson L. M. Gallins P.. (2005). Complement factor H variant increases the risk of age-related macular degeneration. Science 308, 419–421. 10.1126/science.1110359 15761120

[B32] Hariri A. R. Mattay V. S. Tessitore A. Kolachana B. Fera F. Goldman D.. (2002). Serotonin transporter genetic variation and the response of the human amygdala. Science 297, 400–403. 10.1126/science.1071829 12130784

[B33] Hecht-Nielsen R. (1994). Context Vectors: General Purpose Approximate Meaning Representations Self-Organized from Raw Data. Computational Intelligence: Imitating Life. Psciataway, NJ: USAIEEE Press.

[B34] Hibar D. P. Stein J. L. Kohannim O. Jahanshad N. Jack C. R. Weiner M. W. (2011a). Principal components regression: multivariate, gene-based tests in imaging genomics, in IEEE International Symposium on Biomedical Imaging: From Nano to Macro (Chicago, IL), 289–293.

[B35] Hibar D. P. Stein J. L. Kohannim O. Jahanshad N. Saykin A. J. Shen L.. (2011b). Voxelwise gene-wide association study (vGeneWAS): multivariate gene-based association testing in 731 elderly subjects. Neuroimage 56, 1875–1891. 10.1016/j.neuroimage.2011.03.077 21497199 PMC3366726

[B36] Hida K. Wada J. Eguchi J. Zhang H. Baba M. Seida A.. (2005). Visceral adipose tissue-derived serine protease inhibitor: a unique insulin-sensitizing adipocytokine in obesity. Proc. Natl. Acad. Sci. U.S.A. 102, 10610–10615. 10.1073/pnas.0504703102 16030142 PMC1180799

[B37] Hotelling H. (1936). Relations between two sets of variates. Biometrika 28, 321–377.

[B38] Hua W. Y. Nichols T. E. Ghosh D. the Alzheimer's Disease Neuroimaging Initiative (2015). Multiple comparison procedures for neuroimaging genomewide association studies. Biostatistics 16, 17–30. 10.1093/biostatistics/kxu026 24963012 PMC4263222

[B39] Indyk P. Motwani R. (1998). Approximate nearest neighbors: towards removing the curse of dimensionality, in Proceedings of the 30th Annual ACM Symposium on Theory of Computing (Dallas, TX), 604–613.

[B40] Jansen A. G. Mous S. E. White T. Posthuma D. Polderman T. J. C. (2015). What twin studies tell us about the heritability of brain development, morphology, and function: a review. Neuropsychol. Rev. 25, 27–46. 10.1007/s11065-015-9278-9 25672928 PMC4412550

[B41] Johnson W. B. Lindenstrauss J. (1984). Extensions of Lipschitz mappings into a Hilbert space. Proc. Conf. Mod. Anal. Probab. 26, 189–206.

[B42] Kaski S. (1998). Dimensionality reduction by random mapping: fast similarity computation for clustering. Proc. IEEE Int. Joint Conf. Neural Netw. 1, 413–418.

[B43] Le Floch E. Guillemot V. Frouin V. Pinel P. Lalanne C. Trincherah L.. (2012). Significant correlation between a set of genetic polymorphisms and a functional brain network revealed by feature selection and sparse Partial Least Squares. Neuroimage 63, 11–24. 10.1016/j.neuroimage.2012.06.061 22781162

[B44] LeDoux J. (2007). The amygdala. Curr. Biol. 17, 868–874. 10.1016/j.cub.2007.08.005 17956742

[B45] Lee B. T. Ham B. J. (2008). Monoamine oxidase A-uVNTR genotype affects limbic brain activity in response to affective facial stimuli. Neuroreport 19, 515–519. 10.1097/WNR.0b013e3282f94294 18388730

[B46] Li J. Chen Y. (2008). Generating samples for association studies based on HapMap data. BMC Bioinform. 9, 1–13. 10.1093/bib/bbm058 18218094 PMC2375120

[B47] Lin J. Gunopulos D. (2003). Dimensionality reduction by random projection and latent semantic indexing, in Proceedings of the 3rd SIAM International Conference on Data Mining (San Francisco, CA).

[B48] Liu L. Fieguth P. W. (2012). Texture classification from random features. IEEE Trans. Pattern Anal. Mach. Intell. 34, 574–586. 10.1109/TPAMI.2011.145 21768653

[B49] McIntosh A. R. Bookstein F. L. Haxby J. V. Grady C. L. (1996). Spatial pattern analysis of functional brain images using Partial Least Squares. Neuroimage 3, 143–157. 9345485 10.1006/nimg.1996.0016

[B50] Menon A. K. (2007). Random Projections and Applications to Dimensionality Reduction. Honours thesis, The University of Sydney.

[B51] Meyer-Lindenberg A. Weinberger D. R. (2006). Intermediate phenotypes and genetic mechanisms of psychiatric disorders. Nat. Rev. Neurosci. 7, 818–827. 10.1038/nrn1993 16988657

[B52] Ousdal O. T. Brown A. A. Jensen J. Nakstad P. H. Melle I. Agartz I.. (2012). Association between variants near a monoaminergic pathway gene (PHOX2B) and amygdala reactivity: a genome-wide functional imaging study. Twin Res. Hum. Genet. 15, 273–285. 10.1017/thg.2012.5 22856363

[B53] Palmer A. D. Bunch J. Styles I. B. (2015). The use of random projections for the analysis of mass spectrometry imaging data. J. Am. Soc. Mass Spectrom. 26, 315–322. 10.1007/s13361-014-1024-7 25522725 PMC4320302

[B54] Papadimitriou C. H. Raghavan P. Tamaki H. Vempala S. (1998). Latent semantic indexing: a probabilistic analysis, in Proceedings of the 17th Annual Symposium on Principles of Database Systems (Seattle, WA), 159–168.

[B55] Pearson K. (1901). On lines and planes of closest fit to systems of points in space. Philos. Mag. 2, 559–572.

[B56] Pevsner J. (2009). Bioinformatics and Functional Genomics, 2nd Edn. New York, NY: Wiley-Blackwell.

[B57] Plomin R. Owen M. J. McGuffin P. (1994). The genetic basis of complex human behaviors. Science 264, 1733–1739. 8209254 10.1126/science.8209254

[B58] Potkin S. G. Turner J. A. Guffanti G. Lakatos A. Fallon J. H. Nguyen D. D.. (2009). A genome-wide association study of schizophrenia using brain activation as a quantitative phenotype. Schizophr. Bull. 35, 96–108. 10.1093/schbul/sbn155 19023125 PMC2643953

[B59] Purcell S. Neale B. Todd-Brown K. Thomas L. Ferreira M. A. Bender D.. (2007). PLINK: a tool set for whole-genome association and population-based linkage analyses. Am. J. Hum. Genet. 81, 559–575. 10.1086/519795 17701901 PMC1950838

[B60] Schork N. J. Greenwood T. A. Braff D. L. (2007). Statistical genetics concepts and approaches in schizophrenia and related neuropsychiatric research. Schizophr. Bull. 33, 95–104. 10.1093/schbul/sbl045 17035359 PMC2632283

[B61] Schraa-Tam C. K. Rietdijk W. J. Verbeke W. J. Dietvorst R. C. van den Berg W. E. Bagozzi R. P.. (2012). fMRI activities in the emotional cerebellum: a preference for negative stimuli and goal-directed behavior. Cerebellum 11, 233–245. 10.1007/s12311-011-0301-2 21761197 PMC3311856

[B62] Silverman G. A. Bird P. I. Carrell R. W. Church F. C. Coughlin P. B. Gettins P. G.. (2001). The serpins are an expanding superfamily of structurally similar but functionally diverse proteins: evolution, mechanism of inhibition, novel functions, and a revised nomenclature. J. Biol. Chem. 276, 33293–33296. 10.1074/jbc.R100016200 11435447

[B63] Smith S. M. Jenkinson M. Woolrich M. W. Beckmann C. F. Behrens T. E. J. Johansen-Berg H.. (2004). Advances in functional and structural MR image analysis and implementation as FSL. Neuroimage 23, 208–219. 10.1016/j.neuroimage.2004.07.051 15501092

[B64] Strehl A. Ghosh J. (2000). Value-based customer grouping from large retail data-sets, Proceedings of the SPIE Conference on Data Mining and Knowledge Discovery (Kyoto), Vol. 4057, 33–42.

[B65] Sui J. Adali T. Yu Q. Chen J. Calhoun V. D. (2012). A review of multivariate methods for multimodal fusion of brain imaging data. J. Neurosci. Methods 204, 68–81. 10.1016/j.jneumeth.2011.10.031 22108139 PMC3690333

[B66] Sulić V. Perš J. Kristan M. Kovačič S. (2010). Efficient dimensionality reduction using random projection, in Proceedings of the 15th Computer Vision Winter Workshop of the Czech Pattern Recognition Society (Nové Hrady), 29–36.

[B67] Surguladze S. A. Marshall N. Schulze K. Hall M. H. Walshe M. Bramon E.. (2010). Exaggerated neural response to emotional faces in patients with bipolar disorder and their first-degree relatives. Neuroimage 53, 58–64. 10.1016/j.neuroimage.2010.05.069 20595014

[B68] Székely G. J. Rizzo M. L. Bakirov N. K. (2007). Measuring and testing dependence by correlation of distances. Ann. Stat. 35, 2769–2794. 10.1214/009053607000000505

[B69] The HapMap Consortium (2003). The international HapMap project. Nature 426, 789–796. 10.1038/nature02168 14685227

[B70] Tibshirani R. J. Efron B. (1993). An Introduction to the Bootstrap. Monographs on Statistics and Applied Probability. London: Chapman and Hall.

[B71] Tucker L. R. (1958). An inter-battery method of factor analysis. Psychometrika 23, 111–136.

[B72] Vempala S. S. (2004). The Random Projection Method. DIMACS series in discrete mathematics and theoretical computer science, Vol. 65 Providence, RI: American Mathematical Society.

[B73] Wang D. G. Fan J.-B. Siao C.-J. Berno A. Young P. Sapolsky R.. (1998). Large-scale identification, mapping, and genotyping of single-nucleotide polymorphisms in the human genome. Science 280, 1077–1082. 9582121 10.1126/science.280.5366.1077

[B74] Wold H. (1975). Path models with latent variables: the NIPALS approach, in Quantitative Sociology: International Perspectives on Mathematical and Statistical Modeling, eds Blalock H. M. Aganbegian A. Borodkin F. M. Boudon R. Capecchi V. (New York, NY: Academic Press; San Francisco; London), 307–357.

