# Supplementary material for: Random Projection for Fast and Efficient Multivariate Correlation Analysis of High-Dimensional Data: A New Approach
Source: Front Genet. 2016 Jun 7;7:102. doi: 10.3389/fgene.2016.00102 (PMC4894907; doi:10.3389/fgene.2016.00102)
Supplement: Supplementary file 1 [file DataSheet1.pdf]

Supplementary Material: Random Projection for fast and efficient multivariate correlation analysis of high-dimensional data: A new approach

Claudia Grellmann*, Jane Neumann, Sebastian Bitzer, Peter Kovacs, Anke Tönjes, Lars T. Westlye, Ole A. Andreassen, Michael Stumvoll, Arno Villringer and Annette Horstmann

*Correspondence:
Claudia Grellmann
grellmann@cbs.mpg.de

1 SUPPLEMENTARY TABLES

Table 1. Average PLSC and PLSC-RP weights for high-dimensional neuroimaging data
The table shows average PLSC weights and average PLSC-RP weights for causal voxels and causal SNPs as compared to non-causal voxels and non-causal SNPs. Causal voxels and causal SNPs receive higher weights than non-causal voxels and SNPs. Average weights are very similar for PLSC and PLSC-RP.

dimensionality of MRI data	PLS analysis	$\|\bar{w}_{\text{MRI}}\|$ for causal voxels	$\|\bar{w}_{\text{MRI}}\|$ for non-causal voxels	$\|\bar{w}_{\text{SNP}}\|$ for causal SNPs	$\|\bar{w}_{\text{SNP}}\|$ for non-causal SNPs
1,000	PLSC	0.0503	0.0256	0.3395	0.0976
	PLSC-RP	0.0506	0.0255	0.3314	0.0995
10,000	PLSC	0.0152	0.0078	0.3863	0.0901
	PLSC-RP	0.0152	0.0078	0.3710	0.0911
20,000	PLSC	0.0116	0.0054	0.3440	0.0934
	PLSC-RP	0.0114	0.0055	0.3266	0.0919
30,000	PLSC	0.0104	0.0044	0.3877	0.0881
	PLSC-RP	0.0104	0.0045	0.3856	0.0898
40,000	PLSC	0.0089	0.0041	0.3663	0.0928
	PLSC-RP	0.0086	0.0041	0.3533	0.0937
50,000	PLSC	0.0084	0.0039	0.3143	0.0993
	PLSC-RP	0.0086	0.0039	0.3123	0.1019
70,000	PLSC	0.0061	0.0033	0.3325	0.1005
	PLSC-RP	0.0058	0.0034	0.3226	0.0973
90,000	PLSC	0.0058	0.0029	0.2873	0.1074
	PLSC-RP	0.0057	0.0029	0.2772	0.1091

Table 2. Average PLSC and PLSC-RP weights for the fMRI face-matching task
Average weights for causal and non-causal voxels and SNPs are very similar for PLSC and PLSC-RP.

PLS analysis	$\lvert\bar{w}_{\text{MRI}}\rvert$ for causal voxels	$\lvert\bar{w}_{\text{MRI}}\rvert$ for non-causal voxels	$\lvert\bar{w}_{\text{SNP}}\rvert$ for causal SNPs	$\lvert\bar{w}_{\text{SNP}}\rvert$ for non-causal SNPs
PLSC	0.0059	0.0017	0.5768	0.0296
PLSC-RP	0.0059	0.0017	0.5757	0.0467

Table 3. Average SNP weights for PLSC and PLSC-RP in the Sorbs
The table shows average PLSC weights and average PLSC-RP weights for causal and non-causal SNPs. In addition, it is illustrated how serum vaspin and body height are weighted in the first component of the phenotype weight profile.

PLS analysis	$\lvert w_{\text{Vaspin}}\rvert$	$\lvert w_{\text{Height}}\rvert$	$\lvert\bar{w}_{\text{SNP}}\rvert$ for causal SNPs	$\lvert\bar{w}_{\text{SNP}}\rvert$ for non-causal SNPs
PLSC	0.7068	0.0285	0.0093	0.0013
PLSC-RP	0.7068	0.0294	0.0093	0.0013

Table 4. Average PLSC and PLSC-RP weights for high-dimensional neuroimaging and high-dimensional SNP data
Causal voxels and causal SNPs receive higher weights than non-causal voxels and SNPs. Average weights are very similar for PLSC and PLSC-RP.

dim. of MRI data	dim. of SNP data	PLS analysis	$\lvert\bar{w}_{\text{MRI}}\rvert$ for causal voxels	$\lvert\bar{w}_{\text{MRI}}\rvert$ for non-causal voxels	$\lvert\bar{w}_{\text{SNP}}\rvert$ for causal SNPs	$\lvert\bar{w}_{\text{SNP}}\rvert$ for non-causal SNPs
1,000	1,000	PLSC	0.0405	0.0288	0.0947	0.0246
		PLSC-RP	0.0435	0.0279	0.0985	0.0245
10,000	10,000	PLSC	0.0158	0.0077	0.0370	0.0080
		PLSC-RP	0.0151	0.0078	0.0368	0.0079
20,000	20,000	PLSC	0.0116	0.0054	0.0256	0.0056
		PLSC-RP	0.0106	0.0056	0.0240	0.0056
40,000	40,000	PLSC	0.0092	0.0041	0.0179	0.0040
		PLSC-RP	0.0092	0.0040	0.0187	0.0040
50,000	1,000	PLSC	0.0078	0.0040	0.1073	0.0244
		PLSC-RP	0.0070	0.0039	0.0952	0.0246
1,000	50,000	PLSC	0.0395	0.0290	0.0150	0.0036
		PLSC-RP	0.0361	0.0293	0.0142	0.0036

2 SUPPLEMENTARY EQUATIONS

PLSC-RP for dimensionality reduction in X_1 OR X_2

For traditional PLSC, SVD is used to decompose the cross-product matrix $\boldsymbol{A}$ of $\boldsymbol{X_1}$ and $\boldsymbol{X_2}$, which are both standardized column-wise, into three matrices:

$$\text{cov}(\boldsymbol{X_1}, \boldsymbol{X_2}) = \boldsymbol{A} = \boldsymbol{X_1^{'}} \boldsymbol{X_2} = \boldsymbol{W_1} \boldsymbol{S} \boldsymbol{W_2^{'}}. \tag{1}$$

Assumed that $\boldsymbol{X_1}$ is high-dimensional, RP transforms $\boldsymbol{X_1}$ to a lower dimensional space via the following transformation:

$$\boldsymbol{X_{1_{\text{RP}}}} = \boldsymbol{X_1} \cdot \boldsymbol{R}, \tag{2}$$

where $\boldsymbol{R}$ is a random matrix and $\boldsymbol{X_{1_{\text{RP}}}}$ is the low-dimensional subspace of $\boldsymbol{X_1}$ with desired lower dimension k. If we perform PLSC to decompose the cross-product matrix of $\boldsymbol{X_{1_{\text{RP}}}}$ and $\boldsymbol{X_2}$, we obtain the weights $\boldsymbol{W_2}$ for data set $\boldsymbol{X_2}$, but weights $\boldsymbol{W_{1_{\text{RP}}}}$ for the reduced data set $\boldsymbol{X_{1_{\text{RP}}}}$. To transform the weights $\boldsymbol{W_{1_{\text{RP}}}}$ back to the original space, that is $\boldsymbol{W_1}$, we rearrange the equation for the SVD as follows:

Starting point for the rearrangement: the PLSC equation

$$\text{cov}(\boldsymbol{X_1}, \boldsymbol{X_2}) = \boldsymbol{A} = \boldsymbol{X_1^{'}} \boldsymbol{X_2} = \boldsymbol{W_1} \boldsymbol{S} \boldsymbol{W_2^{'}}.$$

If we extend both sides of the equation by $\boldsymbol{w_{2_i}}$, we obtain

$$\boldsymbol{A} \cdot \boldsymbol{w_{2_i}} = \boldsymbol{W_1} \boldsymbol{S} \boldsymbol{W_2^{'}} \cdot \boldsymbol{w_{2_i}}.$$

Since $\boldsymbol{W_2}$ is column-wise orthogonal, we have

$$\boldsymbol{A} \cdot \boldsymbol{w_{2_i}} = \boldsymbol{w_{1_i}} \boldsymbol{s_i} \boldsymbol{w_{2_i}^{'}} \cdot \boldsymbol{w_{2_i}}.$$

Rearranging yields

$$\frac{1}{s_i} \cdot \boldsymbol{A} \cdot \boldsymbol{w_{2_i}} = \boldsymbol{w_{1_i}} \cdot \boldsymbol{w_{2_i}^{'}} \cdot \boldsymbol{w_{2_i}}.$$

Since the L2-norm for a vector $\boldsymbol{a}$ is given by

$$|\boldsymbol{a}| = \sqrt{\boldsymbol{a_1^2} + \boldsymbol{a_2^2} + \ldots + \boldsymbol{a_n^2}},$$
$$|\boldsymbol{a}|^2 = \boldsymbol{a_1^2} + \boldsymbol{a_2^2} + \ldots + \boldsymbol{a_n^2},$$

we obtain the weights $\boldsymbol{w_{1_i}}$, $i = 1, \ldots, p$, $p = \min(k, d_2)$, as follows:

$$\boldsymbol{w_{1_i}} = \frac{1}{s_i \cdot |\boldsymbol{w_{2_i}}|^2} \cdot \boldsymbol{A} \cdot \boldsymbol{w_{2_i}}. \tag{3}$$

PLSC-RP for dimensionality reduction in $\boldsymbol{X_1}$ AND $\boldsymbol{X_2}$

Assumed that both $\boldsymbol{X_1}$ and $\boldsymbol{X_2}$ are high-dimensional, RP transforms $\boldsymbol{X_1}$ and $\boldsymbol{X_2}$ to lower dimensional spaces via the following transformation:

$$\begin{aligned} \boldsymbol{X_1}_{\mathrm{RP}} &= \boldsymbol{X_1} \cdot \boldsymbol{R_1}, \\ \boldsymbol{X_2}_{\mathrm{RP}} &= \boldsymbol{X_2} \cdot \boldsymbol{R_2}. \end{aligned} \tag{4}$$

If we perform PLSC to decompose the cross-product matrix of $\boldsymbol{X_1}_{\mathrm{RP}}$ and $\boldsymbol{X_2}_{\mathrm{RP}}$, we obtain weights $\boldsymbol{W_1}_{\mathrm{RP}}$ and $\boldsymbol{W_2}_{\mathrm{RP}}$ for the low dimensional subspaces. To transform the weights $\boldsymbol{W_1}_{\mathrm{RP}}$ back to the original space $\boldsymbol{W_1}$, we rearrange the equation for the SVD as follows:

Starting point for the rearrangement: the PLSC equation

$$\mathrm{cov}(\boldsymbol{X_1}, \boldsymbol{X_2}_{\mathrm{RP}}) = \boldsymbol{W_1 S W}'_{\boldsymbol{2}_{\mathrm{RP}}}.$$

If we extend both sides of the equation by $\boldsymbol{w_2}_{\mathrm{RP}_i}$, we obtain

$$\mathrm{cov}(\boldsymbol{X_1}, \boldsymbol{X_2}_{\mathrm{RP}}) \cdot \boldsymbol{w_2}_{\mathrm{RP}_i} = \boldsymbol{W_1 S W}'_{\boldsymbol{2}_{\mathrm{RP}}} \cdot \boldsymbol{w_2}_{\mathrm{RP}_i}.$$

Since $\boldsymbol{W_2}_{\mathrm{RP}}$ is column-wise orthogonal, we have

$$\mathrm{cov}(\boldsymbol{X_1}, \boldsymbol{X_2}_{\mathrm{RP}}) \cdot \boldsymbol{w_2}_{\mathrm{RP}_i} = \boldsymbol{w_1}_i s_i \boldsymbol{w}'_{\boldsymbol{2}_{\mathrm{RP}_i}} \cdot \boldsymbol{w_2}_{\mathrm{RP}_i}.$$

Rearranging yields

$$\frac{1}{s_i} \cdot \mathrm{cov}(\boldsymbol{X_1}, \boldsymbol{X_2}_{\mathrm{RP}}) \cdot \boldsymbol{w_2}_{\mathrm{RP}_i} = \boldsymbol{w_1}_i \cdot \boldsymbol{w}'_{\boldsymbol{2}_{\mathrm{RP}_i}} \cdot \boldsymbol{w_2}_{\mathrm{RP}_i}.$$

Thus, for the weights $\boldsymbol{w_1}_i$, $i = 1, \ldots, p$, $p = \min(k_1, k_2)$, we obtain

$$\boldsymbol{w_1}_i = \frac{1}{s_i \cdot |\boldsymbol{w_2}_{\mathrm{RP}_i}|^2} \cdot \mathrm{cov}(\boldsymbol{X_1}, \boldsymbol{X_2}_{\mathrm{RP}}) \cdot \boldsymbol{w_2}_{\mathrm{RP}_i}. \tag{5}$$

Following the same logic, the weights $\boldsymbol{W_2}_{\mathrm{RP}}$ are transformed back to the original space $\boldsymbol{W_2}$ by rearranging the equation for the SVD as follows:

Starting point for the rearrangement: the PLSC equation

$$\mathrm{cov}(\boldsymbol{X_1}_{\mathrm{RP}}, \boldsymbol{X_2}) = \boldsymbol{W_1}_{\mathrm{RP}} \boldsymbol{S W}'_{\boldsymbol{2}}.$$

If we extend both sides of the equation by $\boldsymbol{w}'_{\boldsymbol{1}_{\mathrm{RP}_i}}$, we obtain

$$\boldsymbol{w}'_{\boldsymbol{1}_{\mathrm{RP}_i}} \cdot \mathrm{cov}(\boldsymbol{X_1}_{\mathrm{RP}}, \boldsymbol{X_2}) = \boldsymbol{w}'_{\boldsymbol{1}_{\mathrm{RP}_i}} \cdot \boldsymbol{W_1}_{\mathrm{RP}} \boldsymbol{S W}'_{\boldsymbol{2}}.$$

Since $\boldsymbol{W}_{\boldsymbol{1}_{\mathrm{RP}}}$ is column-wise orthogonal, we have

$$\boldsymbol{w}_{\boldsymbol{1}_{\mathrm{RP}_i}}^{'} \cdot \mathrm{cov}(\boldsymbol{X}_{\boldsymbol{1}_{\mathrm{RP}}}, \boldsymbol{X}_{\boldsymbol{2}}) = \boldsymbol{w}_{\boldsymbol{1}_{\mathrm{RP}_i}}^{'} \cdot \boldsymbol{w}_{\boldsymbol{1}_{\mathrm{RP}_i}} \cdot s_i \cdot \boldsymbol{w}_{\boldsymbol{2}_i}^{'}.$$

Rearranging yields

$$\frac{1}{s_i} \cdot \boldsymbol{w}_{\boldsymbol{1}_{\mathrm{RP}_i}}^{'} \cdot \mathrm{cov}(\boldsymbol{X}_{\boldsymbol{1}_{\mathrm{RP}}}, \boldsymbol{X}_{\boldsymbol{2}}) = \boldsymbol{w}_{\boldsymbol{1}_{\mathrm{RP}_i}}^{'} \cdot \boldsymbol{w}_{\boldsymbol{1}_{\mathrm{RP}_i}} \cdot \boldsymbol{w}_{\boldsymbol{2}_i}^{'}.$$

Thus, for the weights $\boldsymbol{w}_{\boldsymbol{2}_i}$, $i = 1, \ldots, p$, $p = \min(k_1, k_2)$, we obtain

$$\begin{aligned} \boldsymbol{w}_{\boldsymbol{2}_i}^{'} &= \frac{1}{s_i \cdot |\boldsymbol{w}_{\boldsymbol{1}_{\mathrm{RP}_i}}|^2} \cdot \boldsymbol{w}_{\boldsymbol{1}_{\mathrm{RP}_i}}^{'} \cdot \mathrm{cov}(\boldsymbol{X}_{\boldsymbol{1}_{\mathrm{RP}}}, \boldsymbol{X}_{\boldsymbol{2}}), \\ \boldsymbol{w}_{\boldsymbol{2}_i} &= \frac{1}{s_i \cdot |\boldsymbol{w}_{\boldsymbol{1}_{\mathrm{RP}_i}}|^2} \cdot (\mathrm{cov}(\boldsymbol{X}_{\boldsymbol{1}_{\mathrm{RP}}}, \boldsymbol{X}_{\boldsymbol{2}}))^{'} \cdot \boldsymbol{w}_{\boldsymbol{1}_{\mathrm{RP}_i}}. \end{aligned} \tag{6}$$
